# Chew on this: Oral jaw shape is not correlated with diet type in loricariid catfishes

**DOI:** 10.1371/journal.pone.0277102

**Published:** 2022-11-02

**Authors:** Corinthia R. Black, Jonathan W. Armbruster

**Affiliations:** 1 Department Entomology National Museum of Natural History Smithsonian Institution, Washington, District of Columbia, United States of America; 2 Department of Biological Sciences, Auburn University, Auburn, AL, United States of America; Fundacion Miguel Lillo, ARGENTINA

## Abstract

The correlation between form and function is influenced by biomechanical constraints, natural selection, and ecological interactions. In many species of suction-feeding fishes, jaw shape has shown to be closely associated with diet. However, these correlations have not been tested in fishes that have more complex jaw functions. For example, the neotropical loricariid catfishes possess a ventrally facing oral disk, which allows for the oral jaws to adhere to surfaces to conduct feeding. To determine if jaw shape is correlated to diet type, we assessed oral jaw shape across 36 species using CT scans. Shape was quantified with traditional and automated landmarking in 3DSlicer, and diet type correlation was calculated using the phylogenetic generalized least squares (PGLS) method. We found that traditional and automated processes captured shape effectively when all jaw components were combined. PGLS found that diet type did not correlate to jaw shape; however, there was a correlation between clades with diverse diets and fast evolutionary rates of shape. These results suggest that shape is not constrained to diet type, and that similarly shaped jaws coupled with different types of teeth could allow the fishes to feed on a wide range of materials.

## Introduction

The correlation between morphology and diet has been demonstrated in many animals from fishes to birds [1–4]. In addition to biomechanical constraints and natural selection, ecological interactions, such as consuming prey, are shown to be important drivers of morphological diversification [5–7]. For example, the lower pharyngeal jaw in neotropical cichlids has diversified with different diet types. Burress [8] found that thinner, more gracile-like pharyngeal jaws typically correlate with species that consume soft-bodied organisms like microscopic zooplankton. In contrast, hypertrophied pharyngeal jaws are found in species that consume hard prey, such as mollusks with thick shells. Pharyngeal jaws are hypothesized to be functionally decoupled from the oral jaws, as they are responsible for the processing of food items, whereas the oral jaws are primarily involved in prey capture [9, 10]. In most fishes, the oral jaw quickly opens to create a negative pressure gradient that propels water and prey items into the mouth [11, 12]. Because the jaws are not in direct contact with the food items, it is widely believed that oral jaws are optimized for speed, whereas the pharyngeal jaw is optimized for power [10]. However, there are many fishes that do not use their oral jaws in suction feeding, but instead the oral jaws come in direct contact with food items. For example, some reef fishes use a combination of biting and suction feeding to remove prey items from surfaces, placing different functional requirements on their oral jaw anatomy [13–18]. Direct contact between the oral jaws and the substance being fed upon has likely occurred multiple times in fishes, and this has changed the evolutionary pressures on the jaws leading to a diversity of forms [19].

**Fig 1:**
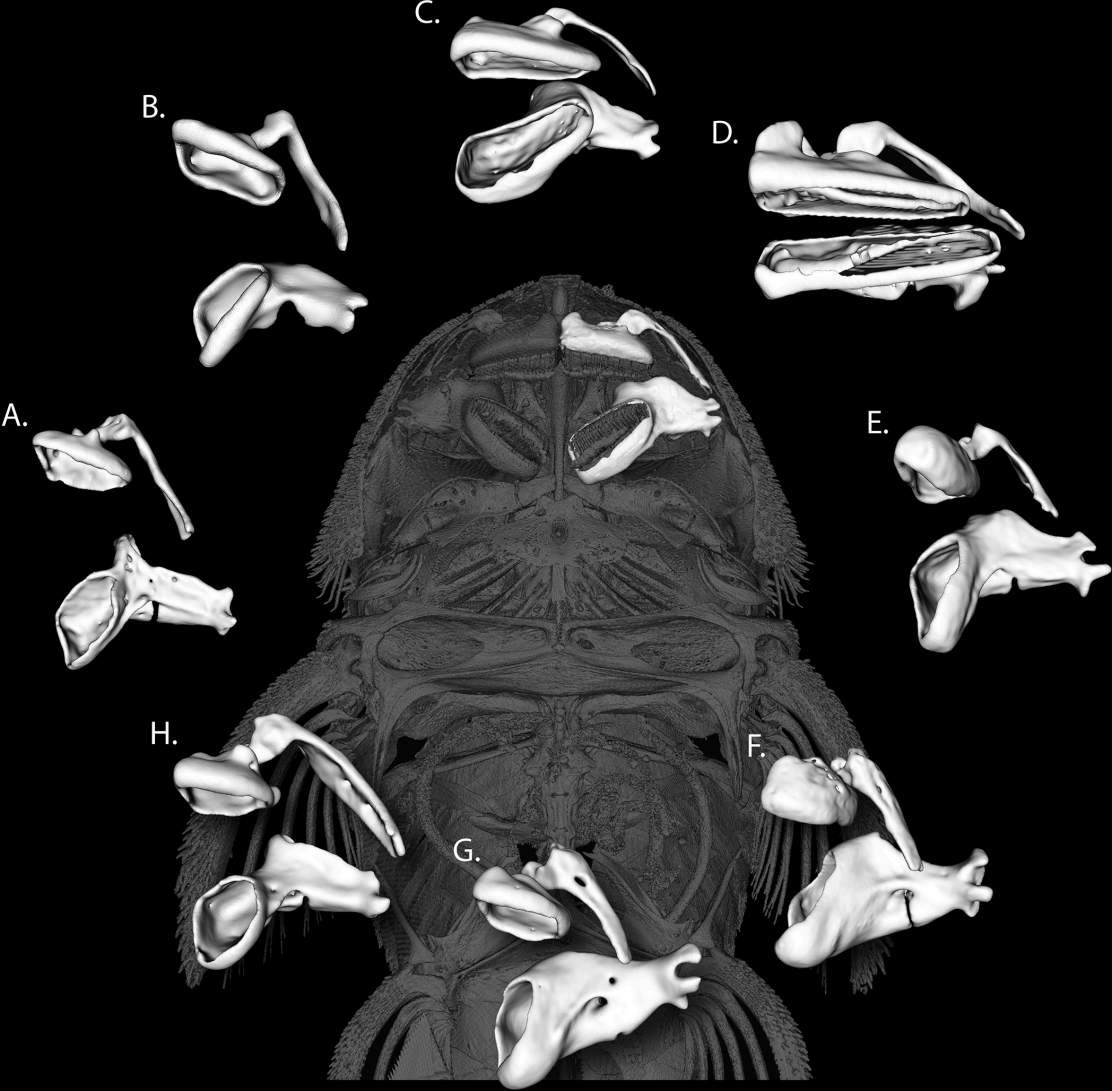
Oral jaw shape diversity in loricariid catfishes. The left oral jaws are shown in white. In the center, the placement of the oral jaws are shown for *Baryancistrus xanthellus*, MorphoSource ID: M37989-69325, Collection ID: ANSP 198216. Representatives of other species are as listed; A) *Otocinclus vittatus*, M39000-70695, ANSP 174732, B) *Farlowella acus*, M37599-68848, ANSP 191381, C) *Harttia loricariformis*, M44340-80321, ANSP 190961, D) *Chaetostoma milesi*, M24790-48802, AUM Unknown ID, E) *Panaqolus*, M37593-68839, ANSP 194642, F) *Hypancistrus vandragti*, M37980-69316, ANSP 199002, G) *Hypancistrus zebra*, M37572-68813, ANSP 197839, and H) *Microlepidogaster perforates*, M37975-69310, ANSP 174117. All CT scans were obtained from MorphoSource.

One group of fishes that attaches their oral jaws directly to surfaces to scrape at food particles are the suckermouth armored catfishes, family Loricariidae [20–22]. This large and diverse family of neotropical catfishes consists of over 1000 recognized species and are identified by ossified dermal plates that cover the body and a ventrally located oral disk [23–25]. The oral disk adheres highly mobile and tooth-bearing oral jaws to a surface where the jaws are abducted ~180°, then adducted rostrocaudally to scrape, gouge, and pry at a variety of benthic food items [23]. Unlike other catfishes, the premaxilla in loricariids is highly mobile and is controlled by the maxillary motion via a unique branch of the adductor mandibulae [20]. The lower jaw is comprised of medially separated mandibles that rotate around the long axis within a shallow socket at the anteroventral articulation of the quadrate [26]. Most loricariids are assumed to consume an indistinguishable mix of detritus and algae, however some lineages have specialized diets and feed on wood (*Hypostomus cochliodon* species group, *Panaqolus*, and *Panaque*), seeds, and macroinvertebrates [23, 27]. Jaw morphologies are varied and range from robust jaws that are used for consuming wood, to small jaws with long thin teeth used to probe crevices for insects, to very long jaws that likely either gouge algae or winnow materials from amongst filamentous algae (Fig 1). Teeth also vary from less than 10 to greater than 200 and from thin, villiform teeth to long, stout, probing teeth, to spoon-shaped, adz-like teeth. Despite the correlation of some morphotypes to dietary specializations, few species-poor studies have examined the convergence of jaw shape and diet [28–30].

One of the most widely used shape analysis methods to explore the evolution of variation is geometric morphometrics (GM). GM has been integral to understanding how form and function has evolved; however, the increasing complexity of capturing homologous landmarks within a 3D space can lead to researcher biases in shape interpretation. In Lujan and Armbruster [23], the authors attempted to find a single, comparable axis for two-dimensional GM, but various twists in the jaws resulted in no homologous view points across species and standard morphometrics were employed instead. Furthermore, the limited number of homologous landmarks reduces the ability to effectively capture shape in drastically different morphologies, like the oral jaws of loricariids; however, no methods were available at the time to examine these complex structures. Recently designed automated methods allow for the comparison of bones using iterative algorithms to automatically place correspondence points that are effectively homologous to one another across several 3D surface meshes. When compared to traditional 3DGM of primate calcanei, these automated methods produced similar and meaningful shape spaces that avoid researcher errors that are often associated with traditional methods [31]. In this study, we capture the shape of loricariid oral jaws using traditional and automated landmarking methods and test the efficacy of automated methods over traditional. To examine how the shape of the oral jaw evolved, we used phylogenetic comparative methods to test for correlation to diet type, shape relatedness to phylogenic relationships, and evolutionary rates or shape change. We hypothesized that jaw shapes would correlate with diet type, resulting in convergence of shapes.

## Materials and methods

### Data collection

To capture the shape of the oral jaws in the Loricariidae, CT scans from 36 individuals representing 35 species were downloaded from the online repository, Morphosource, and segmented in SlicerMorph, a 3DSlicer toolkit (Table 1). Four subfamilies were represented by the following number of species: Hypoptopomatinae *n =* 14, Hypostominae *n =* 11, and Loricariinae *n =* 10. Because the oral jaws are highly mobile, the left premaxilla, maxilla, and lower jaw were individually isolated, and teeth were removed through segmentations in 3D Slicer to avoid issues with automated landmarking. The surface meshes were saved as PLY files and exported for traditional and automated landmarking processes.

**Table 1:** Specimens used in this study.

Taxon	Abbr. in Fig.	Catalog Number	Morphosource ID/ARK ID	In Roxo et al. (congener)
Loricariidae				
Hypoptopomatinae				
*Gymnotocinclus anosteos*	*G*. *ano*	ANSP187156	M44337-80318	yes
			ark:/87602/m4/M80318	
*Hirtella carinata*	*H*. *car*	ANSP198032	M44346-80328	no
			ark:/87602/m4/M80328	
*Hisonotus maculipinnis*	*H*. *mac*	ANSP187011	M38996-70689	yes (*Hisonotus leucofrenatus* 10881)
			ark:/87602/m4/M70689	
*Hypoptopoma spectabile*	*H*. *spe*	ANSP133767	M38998-70693	yes (*Hypoptopoma psilogaster* 22980)
			ark:/87602/m4/M70693	
*Hypoptopoma thoracatum*	*H*. *tho*	ANSP198923	M37581-68823	yes (*Hypoptopoma thoracanthum* 63837)
			ark:/87602/m4/M68823	
*Microlepidogaster perforatus*	*M*. *per*	ANSP174117	M37975-69310	yes
			ark:/87602/m4/M69310	
*Neoplecostomus microps*	*N*. *mic*	ANSP174122	M37606-68859	yes (*Neoplecostomus franciscoensis* 7208)
			ark:/87602/m4/M68859	
*Otocinclus vittatus*	*O*. *vit*	ANSP174732	M39000-70695	yes
			ark:/87602/m4/M70695	
*Otothyris lophophanes*	*O*. *lop*	ANSP84381	M38995-70687	no
			ark:/87602/m4/M70687	
*Nannoxyropsis ephippia*	*O*. *eph*	ANSP177381	M44347-80330	no
			ark:/87602/m4/M80330	
*Oxyropsis wrightiana*	*O*. *wri*	ANSP193942	M37586-68830	no
			ark:/87602/m4/M68830	
*Pareiorhaphis cameroni*	*P*. *cam*	ANSP173796	M37986-69322	yes (*Pareiorhaphis* sp. 9685)
			ark:/87602/m4/M69322	
*Pareiorhina rudolphi*	*P*. *rud*	ANSP174125	M37990-69326	yes
			ark:/87602/m4/M69326	
*Parotocinclus maculicauda*	*P*. *mac*	ANSP168971	M44336-80317	yes (*Parotocinclus* cf. *bahiensis* 34692)
			ark:/87602/m4/M80317	
Hypostominae				
*Baryancistrus xanthellus*	*B*. *xan*	ANSP198216	M37989-69325	yes (*Baryancistrus beggini* 39227)
			ark:/87602/m4/M69325	
*Chaetostoma milesi*	*C*. *mil*	AUMXXXXX	M24790-48802	yes (*Chaetostoma jegui*)
			ark:/87602/m4/M48802	
*Cordylancistrus torbesensis*	*C*. *tor*	MCZ36170	M24040-47256	no
			ark:/87602/m4/M47256	
*Dekeyseria pulcher*	*D*. *pul*	ANSP185289	M37978-69314	no
			ark:/87602/m4/M69314	
*Hypancistrus zebra*	*H*. *zeb*	ANSP197839	M37572-68813	yes (*Hypancistrus* sp. 61759)
			ark:/87602/m4/M68813	
*Leporacanthicus joselimai*	*L*. *jos*	AUMXXXXX	M24526-48318	yes
			ark:/87602/m4/M48318	
*Lithoxus lithoides*	*L*. *lit*	ANSP177363	M37594-68841	no
			ark:/87602/m4/M68841	
*Micracanthicus vandragti*	*M*. *van*	ANSP199002	M37980-69316	yes
			ark:/87602/m4/M69316	
*Panaqolus sp*.	*P*. *sp*.	ANSP194642	M37593-68839	yes (*Panaqolus* sp. 61753)
			ark:/87602/m4/M68839	
*Panaque nigrolineatus*	*P*. *nig*	ANSP128682, AUMXXXXX	M37982-69318	yes (*Panaque cochliodon* 19170)
			ark:/87602/m4/M69318	
			M24791-48803	
			ark:/87602/m4/M48803	
*Pseudolithoxus tigris*	*P*. *tig*	YPM023896	M44257-80205	yes
			ark:/87602/m4/M80205	
Loricariinae				
*Dentectus barbarmatus*	*D*. *bar*	ANSP160860	M37577-68818	no
			ark:/87602/m4/M68818	
*Farlowella acus*	*F*. *acu*	ANSP191381	M37599-68848	yes (*Farlowella amazona* 26397)
			ark:/87602/m4/M68848	
*Harttia loricariformis*	*H*. *lor*	MCZ8121	M23563-46120	yes
			ark:/87602/m4/M46120	
*Harttiella longicauda*	*H*. *lon*	ANSP190961	M44340-80321	no
			ark:/87602/m4/M80319	
*Hemiodontichthys acipenserinus*	*H*. *aci*	ANSP192919	M37597-68845	no
			ark:/87602/m4/M68845	
*Lamontichthys filamentosus*	*L*. *fil*	ANSP181100	M37571-68812	yes
			ark:/87602/m4/M68812	
*Loricaria clavipinna*	*L*. *cla*	ANSP178472	M37573-68814	yes
			ark:/87602/m4/M69313	
*Loricariichthys maculatus*	*L*. *mac*	ANSP131614	M37590-68835	yes (*Loricariichthys* sp. 22778)
			ark:/87602/m4/M68835	
*Planiloricaria cryptodon*	*P*. *cry*	ANSP191512	M37604-68856	yes
			ark:/87602/m4/M68856	
*Rhadinoloricaria macromystax*	*R*. *mac*	TCWC15249.01	M30925-59120	no
			ark:/87602/m4/M59120	

**Fig 2:**
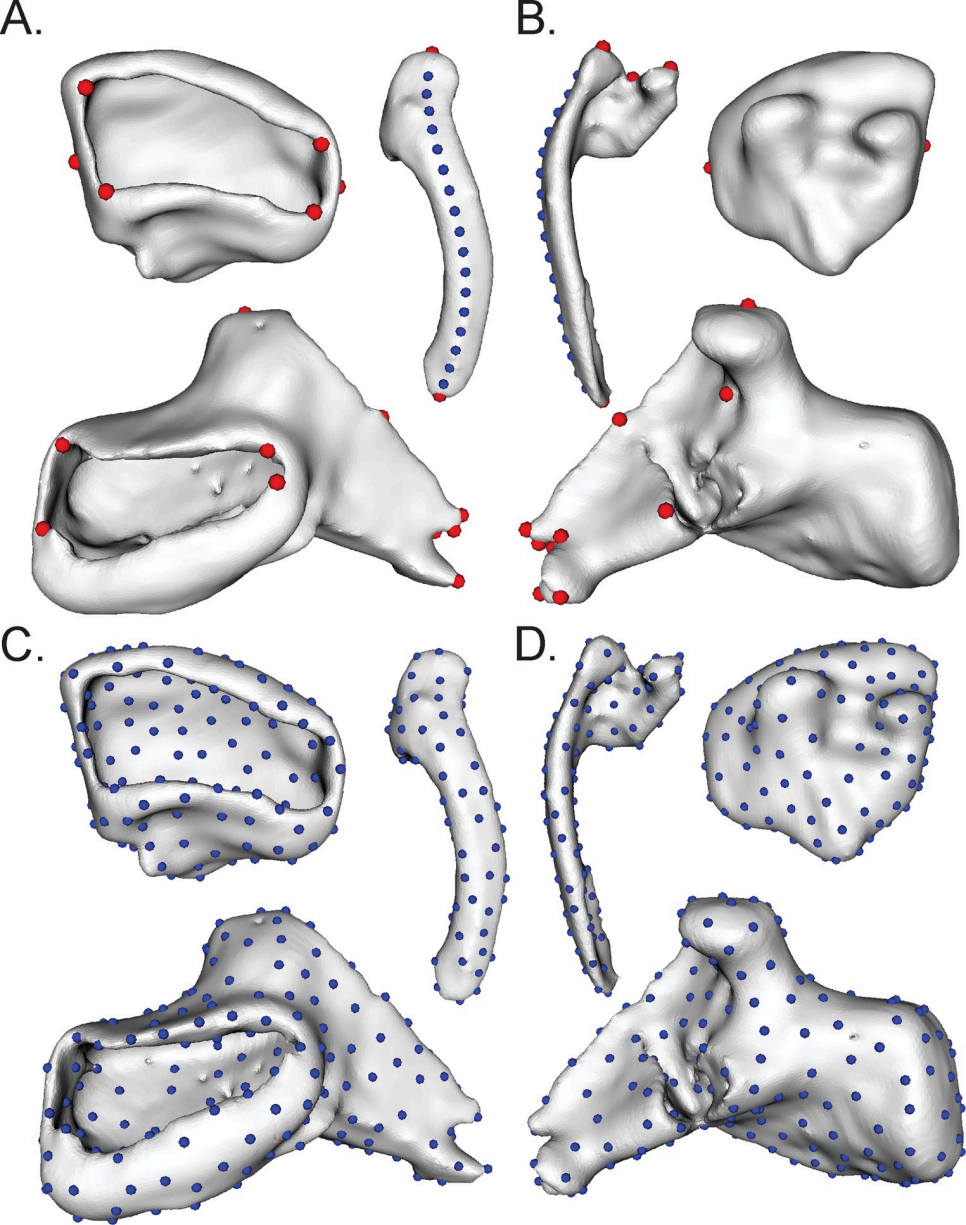
Traditional landmarks in the (A) ventral and (B) dorsal view (red dots homologous, landmarks, blue dots sliding landmarks), and automated landmarks in the (C) ventral and (D) dorsal view as shown on *Gymnotocinclus anosteos*.

Traditional landmarks were captured in SlicerMorph [32] for each skeletal element (Fig 2A and 2B). Premaxillary shape was summarized with six landmarks, denoting the corners of the tooth cup, the most lateral edge of the premaxilla, and the most medial edge of the premaxilla. Maxillary shape was summarized with four landmarks and one curve (20 sliding landmarks), which captured changes in the articulations of the head, the most distal end of the body, and the curvature of the body. Lower jaw shape was summarized by 14 landmarks that denote changes in the tooth cup, height of the crest, articulations of the anguloarticular at the quadrate, and the adductor mandibulae fossa.

Automated pseudolandmarking was completed in the Auto3DGM extension in 3DSlicer [32–34]. Auto3DGM is a homology-free landmarking protocol that places landmarks across the surface of a 3D mesh then uses iterative processes to align meshes to one another so pseudolandmarks are effectively homologous. Shape is represented by 200 pseudolandmarks on the premaxilla, 100 for the maxilla, and 300 for the lower jaw using 1000 iterations for each element (Fig 2C and 2D). The lower jaw consists of two bones, the dentary and anguloarticular. The two bones partially form a suture (sometimes the bones are ankylosed) and partially form a synchondrosis with a cartilaginous section of variable size. These characteristics made it impossible to separate the two bones, and because they operate as a single functional unit, we analyzed the entire lower jaw together. Aligned meshes were imported into Slicermorph to visually check if alignments were correct for automated datasets. Meshes which did not align properly were removed from the dataset. In the premaxilla, six specimens (*Hemiodontichthys acipenserinus*, *Leporacanthicus joselimai*, *Lithoxus lithoides*, *Loricaria clavipinna*, *Loricariichthys maculatus*, and *Planiloricaria cryptodon*) were removed due to alignment issues and/or lack of a tooth cup. All meshes aligned properly for the maxilla and lower jaw elements. Code and raw data available on github at https://github.com/corinthiablack/loricariid-oral-jaw-shape.git.

### Analyses

For each jaw element (premaxilla, maxilla, and the lower jaw) the traditional landmarks and automated pseudolandmarks were superimposed for each dataset in the R package, geomorph, using generalized least squares Procrustes superimposition (GPA) resulting in six data sets [35]. Principal component analyses (PCA) were performed for each dataset and theoretical shapes were determined by warping the 3D surface mesh of mean shape to the extremes of the axes. To obtain a mean surface mesh, we found the mean specimen using findMeanSpec in geomorph and used warpRefMesh to warp the surface mesh to the mean shape of the GPA. Once the mean surface mesh was generated, we used warpRefMesh to warp the mean mesh to the extremes of significant axes for each set. To determine the overall shape variation for the oral jaws, the Procrustes aligned landmarks for all jaw elements were combined using the function combine.subsets for the traditional landmark and automated pseudolandmark data sets in geomorph [36]. We used this method in place of analyzing the entire jaw in vivo because the jaws are highly mobile and can change position during preservation.

To explore evolutionary trends of jaw shape within the Loricariidae, we generated a phylomorphospace by projecting the phylogeny onto the multivariate space for each dataset [37]. Only one species had multiple specimens for which the Procrustes landmarks were averaged. The phylogenomic tree [38] was trimmed to represent the species or their congener in the multivariate dataset in the R package ape (Table 1) [39, 40]. Significant axes for each dataset were found using the broken stick method in the R package PCDimension [41–44]. To identify the shape changes related to phylogenetic signal, we performed a phylogenetically-aligned component analysis (PACA) for all datasets. Unlike phylogenetic PCA, PACA aligns shape data to the axis of greatest phylogenetic signal, maximizing the shape variation related to the phylogeny in the first component to reduces errors in phylogenetic signal and maximize evolutionary rates along the first component [45].

To compare traditional to automated data sets we performed a Partial Least Squares (PLS) regression on each phylomorphospace dataset (combined, premaxilla, maxilla, and lower jaw). The phylogenetic signal was calculated for the total shape and significant axes determined by broken stick method for both PCA and PACA datasets using the K_mult_ method in geomorph. The K_mult_ method uses a Brownian motion model to evaluate the degree of phylogenetic signal in a dataset [35, 46, 47]. Diet type was collected from the literature and phylogenetic generalized least squares (PGLS) were performed in geomorph using the procD.pgls and pairwise functions [48]. PGLS calculates the probability that shape variation is attributed to ecological factors in a linear model. A linear model (shape coordinates ~ diet) was used to detect relationships between shape and diet. For all PGLS tests, a randomized residual permutation procedure with 1,000 permutations was used.

Evolutionary rates for the PACA of combined automated landmarks were calculated for significant axes using a penalized-likelihood model in the R package, phytools [49, 50]. This method calculates evolutionary rates under a Brownian model using a penalty term equal to the log-transformed probability density and is multiplied by a smoothing coefficient (λ). An intermediate λ (λ = 1) was used to give equal weights to probabilities. Ancestral state reconstruction of diet type was calculated under a Brownian motion model in the R package phytools [49].

## Results

### (A) Substantial morphological diversity across species

#### Morphospaces

(See supplemental material for results of individual elements (S1–S3 Figs)). To visualize the total shape variation of the oral jaws, the combined shape was found for the premaxilla, maxilla, and lower jaw. Two axes were significant for traditional landmarks. On PC1, oral jaws with elongate tooth cups, shorter anguloarticulars, and larger maxillary heads were placed on the negative end, whereas oral jaws with shorter tooth cups, elongate anguloarticulars, and smaller maxillary heads were placed on the positive end (S4A and S5–S7 Figs). Along PC2, the bodies of the maxilla and lower jaws were slenderer and became more robust toward the positive end (S4A and S5–S7 Figs).

For automated landmarks, three significant axes were identified through the broken stick method. Along PC1, premaxillary tooth cups were more elongate, processes on the premaxilla and lower jaw were smaller, and the maxilla was slimmer toward the negative end of the axis. On the positive end of the axis, premaxillary tooth cups were shorter, the premaxilla and lower jaw had larger processes, and the maxilla was more robust (S4B and S5–S7 Figs). Across the PC2 axis, premaxillas with elongate tooth cups, smaller maxillary heads, and slenderer anguloarticulars fell on the negative end, whereas on the positive end premaxillas had shorter tooth cups, maxillary heads were larger, and the anguloarticular was more robust (S4B and S5–S7 Figs). The third principal component described only changes in the maxilla and premaxilla, where thinner maxillas and premaxillas with larger processes were placed on the negative end and thicker maxillas and premaxillas with smaller processes were placed on the positive end (S5–S8 Figs).

#### Phylomorphospaces

**Fig 3:**
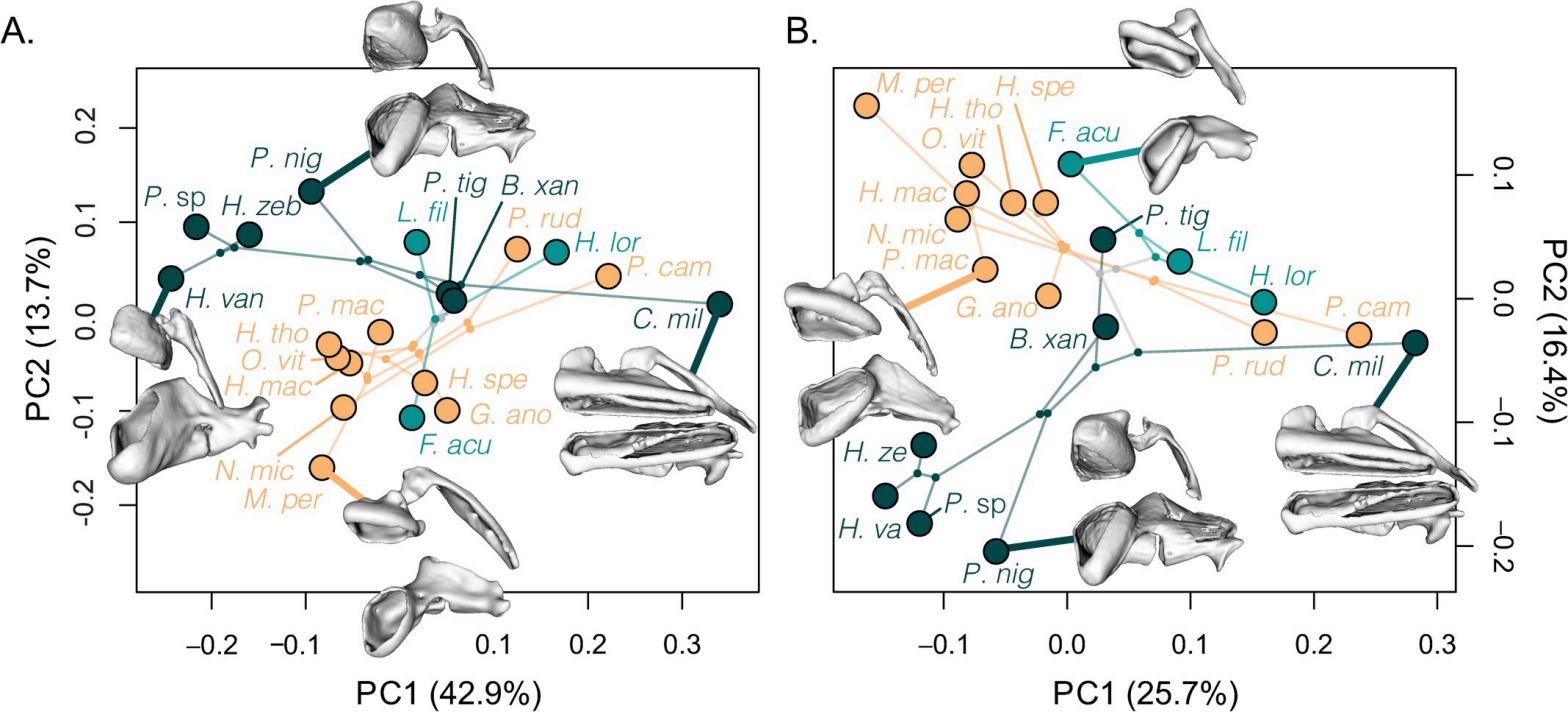
Phylomorphospaces for the combined shape (premaxilla, maxilla, and lower jaw) for loricariid species using (A) traditional and (B) automated landmarking. Subfamilies denoted by colors; Hypoptopomatinae in orange, Hypostominae in dark green, and Loricariinae in light green. See Table 1 for abbreviations of species.

When the morphospace was trimmed to fit the phylogeny [38], the broken stick method found fewer significant axes than the traditional landmarked combined shape dataset (1 significant axis), which suggests some shape data was lost by trimming the dataset to match the phylogenomic data (Fig 3 and S9–S11 Figs). Although some shape data was lost, shape was similar to what was seen in the morphospaces (S12–S14 Figs).

All phylomorphospaces had significant overlap of subfamilies with many instances of convergence within the morphospace. Phylogenetic signal was calculated for the total shape and significant axes of each phylomorphospace dataset. The only dataset that was significant for total shape was the automated landmarking for combined jaw shape with K = 0.428 (*p* = 0.01; S1 Table). When restricted to significant axes, the datasets for traditional landmarking and automated landmarking for the combined jaw shape were significant, with K = 0.9452 (*p* = 0.005) and K = 0.7833 (*p* = 0.001) respectively (S1 Table). To determine if shape was correlated to diet type, PGLS was calculated for all eight datasets, traditional landmarking for premaxilla, maxilla, lower jaw, and combined landmarks, and automated landmarking for premaxilla, maxilla, lower jaw, and combined landmarks. In all cases, shape was not significantly correlated with diet type (S2 Table).

### (B) Automated landmarking preforms better than traditional landmarking methods

To compare how different landmarking schemes preformed to one another, we calculated the PLS between traditional and automated methods (S15 Fig). An r-PLS score of 1 suggests the two datasets are the same, whereas a score of 0 would suggest the datasets are completely different from one another. The traditional and automated landmarks for combined shape showed that traditional and automated landmarking preformed similarly with an r-PLS = 0.942 (*p* = 0.001; S15A Fig). However, when traditional and automated landmarking of individual bones were compared to one another, there was more variation between datasets, with the premaxilla scoring a r-PLS of 0.844 (*p* = 0.005), the maxilla a r-PLS of 0.75 (*p* = 0.032), and the lower jaw a r-PLS of 0.923 (*p* = 0.001; S15B–S15D Fig). In addition to differences between the datasets, the warped meshes for traditional landmarking failed to capture realistic shapes, whereas the automated landmarks produced warped meshes that looked representative of loricariid jaws (S5–S7 and S11–13 Figs).

### (C) Diet type correlates with evolutionary rates

**Fig 4:**
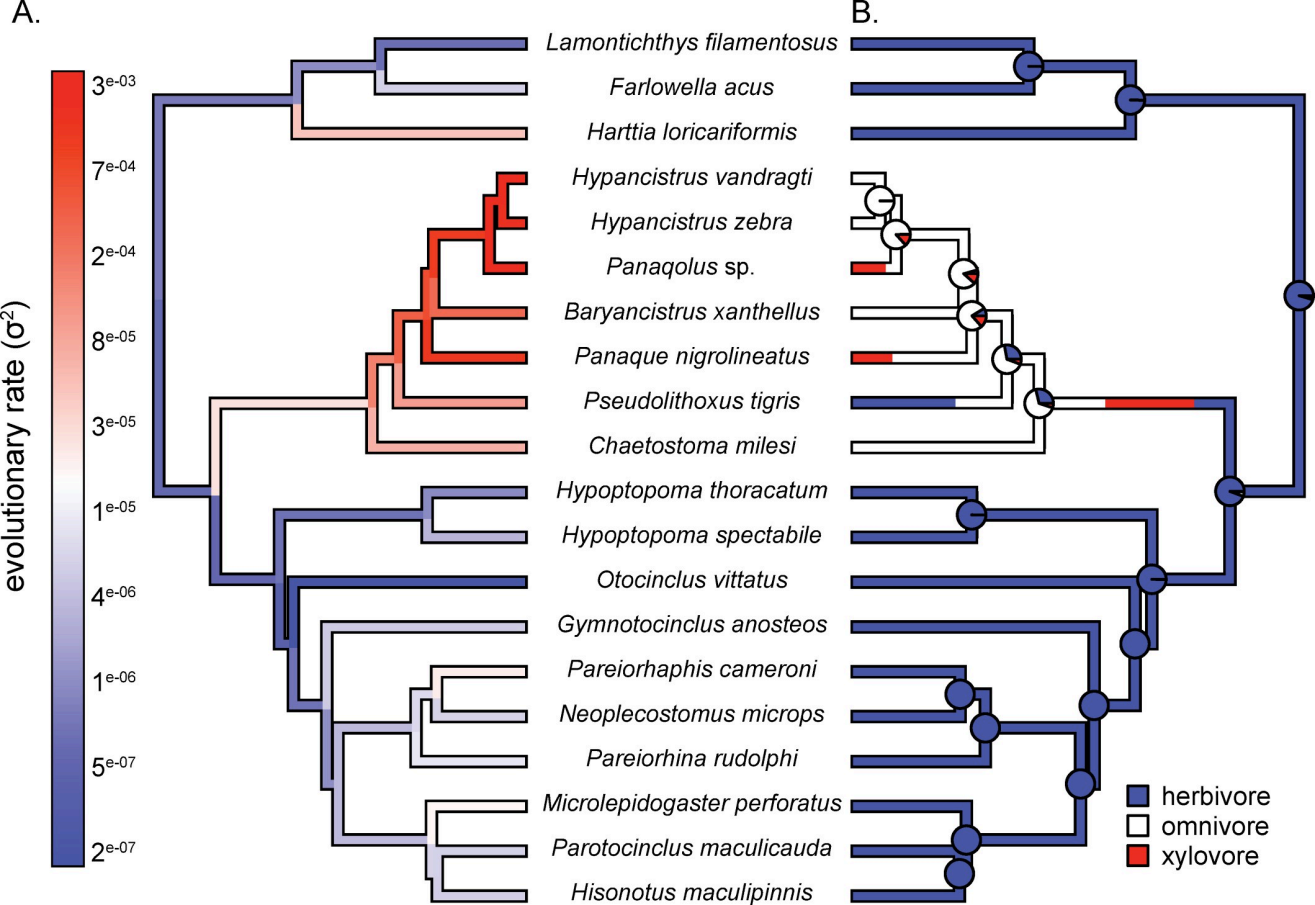
(A) Evolutionary rates for combined dataset of automated landmarks for PC1 using a penalized-likelihood model compared to (B) the ancestral state reconstructions for diet type in loricariid species.

To understand the evolutionary rates of shape change, we calculated evolutionary rates for the PACA on the combined shape data of automated landmarked specimens (Fig 4A and S16 Fig, S3 Table). For evolutionary rates of shape change on the first PC, the fastest evolving species were *Panaque nigrolineatus*, *Panaqolus* sp., and *Micracanthicus vandragti*, with the average of the top three species being 3.6x faster than the average of all species. The slowest species were *Otocinclus vittatus*, *Hypoptopoma thoracatum*, and *Lamontichthys filamentosus*, with the average of the bottom three species being 1x slower than the average of all species (Fig 4A, S3 Table). The fastest evolving species for the second PC were *Neoplecostomus microps*, *Pareiorhaphis cameroni*, and *Panaque nigrolineatus*, with the average of the top three species being 2.7x faster than the average of all species. The slowest species for PC2 were *Parotocinclus maculicauda*, *Farlowella acus*, and *Lamontichthys filamentosus*, with the average of the bottom three species being 1.0x slower than the average of all species (S16 Fig, S3 Table).

To determine how diet type evolved across the loricariids, we calculated ancestral states for primary diet type of each species. The results showed that the most common ancestor of all loricariids were most likely herbivorous and shifted toward omnivores at the base of the subfamily Hypostominae. Within the subfamily Hypostominae, there was one reversal to herbivory and two independent shifts to wood eating (Fig 4B).

## Discussion

This is the first study to use geometric morphometrics to examine the evolution of oral jaw shape in the armored catfishes. In addition to the vast diversity of shape across the species, we found that automated landmarking methods produced more meaningful morphospaces and realistic mesh warps in comparison to traditional landmarks. Shape of the oral jaw does not correlate with phylogeny or diet type, suggesting that neither have an influence on the evolution of the oral jaws in armored catfishes. However, the evolutionary rates of jaw shape correlate with diet type, which may suggest that as loricariids begin to occupy various ecological niches, the oral jaws undergo faster shape changes becoming more disparate in shape. Regardless, the lack of correlation between diet and phylogeny suggests that the loricariid catfishes in our dataset do not have to have specialized jaws for specific diet types. There are some morphologies that are highly adapted to extreme feeding modes (like wood-eating); however, similarly shaped jaws coupled with different types of teeth could allow the fishes to feed on a wide range of materials.

Automated and traditional landmarking schemes show that suckermouth armored catfishes have a wide diversity of oral jaw shapes, with most changes occurring in the tooth cup and anguloarticular length as well as curvature of the maxilla. Within every shape space, there was considerable overlap between the subfamilies. Although morphospaces between automated and traditional processes were similar in variation, there are dramatic differences in placement within shape spaces. PLS shows significant differences between the automated and traditional landmarks, yet the combined dataset of oral jaw elements is surprisingly close between the two methods (r-PLS = 0.942, *p* = 0.001). This may suggest that a single jaw element may not represent the overall jaw shape well. Even in a decoupled system, it is likely that there is a degree of coordinated evolution between the decoupled structures [51]. For example, the premaxilla and lower jaws of *Leporacanthicus joselimai* are very different. *Leporacanthicus joselimai* is a carnivore likely feeding on snails and caddisflies and has been hypothesized to use the lower jaw to hold the prey item and the upper jaw to scoop them out of their shells or cases [52, 53].

Burress and Muñoz [54] found that the pharyngeal jaws can change independently from the oral jaws, but oral jaw changes are always correlated with the pharyngeal jaws. This suggests that capture and processing of food can be decoupled, but the oral jaws cannot be the drivers of such change in cichlids. This has huge implications in functional studies that neglect the oral and pharyngeal jaws as a whole. For example, the biomechanics of loricariid jaw movement has not been successfully modeled and the kinematics are more complex than we have been able to visualize [23, 55]. Loricariids vary in their pharyngeal jaws as well [56] with differences in jaw sizes and shape, tooth number, and tooth size. Seed-feeding loricariids have pharyngeal jaws similar to durophagous cichlids with large, molariform teeth and hypertrophied bones. Future studies should link the oral and pharyngeal jaws together.

In addition to differences in shape spaces, warped 3D meshes were dissimilar between the automated and traditional methods. For extreme warps based on traditional landmarks, the limited number of homologous landmarks produced warps with unnatural shapes, whereas automated processes produced more natural looking warps. This is likely due to the complicated nature of armored catfish oral jaws, which, in addition to articulations and muscle attachments, have complex processes that cannot be captured in traditional landmarking methods. However, automated landmarking methods were limited in the ability to compare wildly different shapes, regardless of the number of intermediates. Boyer and others [31] found that automated landmarking methods were able to align dissimilar objects to one another if constrained by intermediates, however, we found that despite the number of intermediate shapes, certain jaw shapes did not align correctly. For example, most premaxillas are longer mediolaterally, yet a few species have premaxillas that are longer anteroposteriorly. As the premaxillae are simple, automated methods were unable to differentiate between the two, leading to misalignment of shapes that had to be trimmed from the dataset. We were unable to capture extreme shapes, like *L*. *joselimai*, in our datasets as automated methods could not align the premaxillae correctly to other loricariids.

To further complicate things, automated methods were not designed to align fused structures such as the lower jaw. The close association of the dentary to the anguloarticular in the lower jaw made separating the two bones difficult. Because of this, there were some misplacements in the morphospace. *Cordylancistrus torbesensis*, *Pareiorhaphis cameroni*, and *Chaetostoma milesi* have long tooth cups with a short anguloarticular but grouped with *Loricariichthys maculatus* and *Dentectus barbarmatus* which have short tooth cups and a long anguloarticular (S1E and S1F Fig). However, combining the oral jaws into a single dataset seemed to resolve these issues by including the shape of the premaxilla and maxilla with the lower jaw (Fig 3 and S3 Fig). Further research should seek a method that allows automated landmarks to be restricted with traditional landmarks to properly align 3D meshes.

Surprisingly, shape did not correlate with phylogenetic relationships or ecological type. Phylogenetic signal was small or insignificant, which was demonstrated by the notable overlap within the shape space (Fig 3). In previous studies, it was hypothesized that specific diet types correlated with oral jaw shapes. Long jawed loricariids were thought to feed on detritus and algae, whereas loricariids with robust jaws fed on wood [23]. However, our results suggest that there are more complex relationships between jaw shape and diet. PGLS did not correlate shape to diet type, suggesting that a given oral jaw shape may be used to eat a diversity of materials, and likely depends on shape, number, and size of the teeth associated with the jaws as well as the muscles that operate the jaws. Yet, we did find that morphological evolutionary rates correlated with diet type in loricariids, suggesting that as loricariids begin to occupy various ecological niches, the oral jaw shape evolves faster (Fig 4). This is consistent to studies in birds, where it was found that diet and skull shape are not closely associated with one another, nor are beak and diet type, but the morphological evolutionary rates did correlate with diet type [57–59].

Still, inadequate evidence of trophic variability across the loricariids may attribute to the lack of shape correlation found in loricariid jaws. Until recently, trophic partitioning in armored catfishes was based solely on gut content analysis, which is difficult as loricariids have fast passage rates and feed on similar looking material [60, 61]. This study may benefit from modern trophic partitioning methods using stable isotope analyses to refine diet type between species, but comparison between specimens collected as disparate localities can be difficult with stable isotopic analyses [62, 63]. Sequencing gut contents could be another possibility for better establishing what loricariids feed upon, but loricariids consume mostly indigestible material. For example, although wood-eaters consume wood, they are not likely digesting it [64–66]. Determining what is in the gut of a loricariid may not be reflective of what loricariids are digesting and assimilating. Nonetheless, the lack of correlation between jaw shape, phylogeny, and diet type suggests that oral jaw morphology is not constricted to specific diet types. In other words, there are many ways for loricariids to feed on similar and different food items.

What the examination of adaptive radiations has shown us is that modularity is important in establishing morphological diversity. For loricariid jaws, the shape of the jaw itself is only part of the story. Musculature is clearly important, as muscles can change in strength and mechanical advantage allowing for flexibility in feeding with similar jaw forms [23]. Teeth are likely key to ecological partitioning. Similar jaw morphologies were noted between wood-eaters (*Panaque*, *Panaqolus*, and the *Hypostomus cochliodon* group), carnivores (*Leporacanthicus* and *Lithoxus* group), and potential spongivores (*Hypancistrus*) [56]. These taxa have very different teeth suggesting that changing teeth with similar jaw morphologies may allow for accessing different food items. Yet, determining diet type remains a problem with loricariids due to indistinguishable gut contents and the quick gut passage rates that limit the important dietary material in the intestinal track [64–66].

Future work will need to include more taxa, as those sampled here represent a very small swath of the diversity of loricariids, and better resolution CT scans are needed to more adequality capture tooth shape. With these data, teeth could be incorporated into the combined analysis to better parse the shape space. Additionally, the variation in pharyngeal jaws could be incorporated into the dataset. Most pharyngeal jaws are similar across loricariids, nevertheless, there are some specialized pharyngeal jaws in the invertivorous Lithoxini, the granivorous members of the Loricariini, and the algiviorous/detritivrous Rhinelepinae [56, 67]. The examination of diversity of catfish pharyngeal jaws has not been undertaken, and their function in the feeding of loricariid catfishes is unknown.

In this study, we have shown the advantages of 3D automated meshes on complex structures like loricariid jaws, but there are limits in the analysis of the premaxilla due to the simple shape (rectangular box) and in the lower jaw due to the inability to separate the two bones. The function of the loricariid jaw mechanism and its integration with ecology still remains elusive, but it would appear that different jaw morphologies can be used to feed upon different objects depending on the teeth and muscular systems associated with those jaws. This flexibility to use different jaw shapes is likely one thing that supports the great diversity of loricariid species.

## Supporting information

S1 Text Additional results for morphospace analyses. (DOCX)

S1 File (TIF)

S1 Table Significant axes and phylogenetic signal for phylomorphospaces. (XLSX)

S2 Table Phylogenetic generalized least squares for individual and combine oral jaw shape compared to diet type. (XLSX)

S3 Table Evolutionary rates for automated landmarks on combine oral jaw shape for specimens and nodes. (XLSX)

S1 Fig Morphospaces for individual bones using (A) traditional and (B) automated landmarks on the premaxilla, (C) traditional and (D) automated landmarks on the maxilla, and (E) traditional and (F) automated landmarks on the lower jaw. Subfamilies denoted by colors; Hypoptopomatinae in orange, Hypostominae in dark green, Loricariinae in light green, and Neoplecostominae in pink.
(PDF)

S2 Fig Morphospace of the maxilla using traditional landmarks for PC1 and PC3. Subfamilies denoted by colors; Hypoptopomatinae in orange, Hypostominae in dark green, Loricariinae in light green, and Neoplecostominae in pink.
(PDF)

S3 Fig Morphospace for the lower jaw using (A) traditional landmarks and (B) automated landmarking methods for PC1 and PC3. Subfamilies denoted by colors; Hypoptopomatinae in orange, Hypostominae in dark green, Loricariinae in light green, and Neoplecostominae in pink.
(PDF)

S4 Fig Morphospaces for the combine shape (premaxilla, maxilla, and lower jaw) for loricariid species using (A) traditional and (B) automated landmarking. Subfamilies denoted by colors; Hypoptopomatinae in orange, Hypostominae in dark green, Loricariinae in light green, and Neoplecostominae in pink.
(PDF)

S5 Fig Warped 3D meshes for extreme shapes for the combine morphospace of the premaxilla. (PDF)

S6 Fig Warped 3D meshes for extreme shapes for the combine morphospace of the maxilla. (PDF)

S7 Fig Warped 3D meshes for extreme shapes for the combine morphospace of the lower jaw. (PDF)

S8 Fig Morphospace of PC1 and PC3 for the combine shape (premaxilla, maxilla, and lower jaw) using automated landmarking. Subfamilies denoted by colors; Hypoptopomatinae in orange, Hypostominae in dark green, Loricariinae in light green, and Neoplecostominae in pink.
(PDF)

S9 Fig Phylomorphospaces for individual bones using (A) traditional and (B) automated landmarks on the premaxilla, (C) traditional and (D) automated landmarks on the maxilla, and (E) traditional and (F) automated landmarks on the lower jaw. Subfamilies denoted by colors; Hypoptopomatinae in orange, Hypostominae in dark green, and Loricariinae in light green.
(PDF)

S10 Fig Phylomorphospace of PC1 and PC3 for the maxilla using traditional landmarks. Subfamilies denoted by colors; Hypoptopomatinae in orange, Hypostominae in dark green, and Loricariinae in light green.
(PDF)

S11 Fig Phylomorphospace for the combine shape (premaxilla, maxilla, and lower jaw) of loricariid species using automated landmarking. Subfamilies denoted by colors; Hypoptopomatinae in orange, Hypostominae in dark green, and Loricariinae in light green.
(PDF)

S12 Fig Warped 3D meshes for extreme shapes for the combine phylomorphospace of the premaxilla. (PDF)

S13 Fig Warped 3D meshes for extreme shapes for the combine phylomorphospace of the maxilla. (PDF)

S14 Fig Warped 3D meshes for extreme shapes for the combine phylomorphospace of the lower jaw. (PDF)

S15 Fig Partial least squares (PLS) comparing the traditional landmarking skeme to automated landmarking methods for (A) combine oral jaw shape, (B) the premaxilla, (C) the maxilla, and (D) the lower jaw for loricariid catfishes. Subfamilies denoted by colors; Hypoptopomatinae in orange, Hypostominae in dark green, and Loricariinae in light green.
(PDF)

S16 Fig Evolutionary rates of phylogenetically aligned components (PACA) for combine dataset of automated landmarks along using a penalized-likelihood model. (PDF)
