# Supplementary material for: Chew on this: Oral jaw shape is not correlated with diet type in loricariid catfishes
Source: PLoS One. 2022 Nov 2;17(11):e0277102. doi: 10.1371/journal.pone.0277102 (PMC9629652; doi:10.1371/journal.pone.0277102)
Supplement: S1 Text — (DOCX) [file pone.0277102.s001.docx]

**Supplemental Text**

**Results**

Morphospaces. All shape analyses show complex variation in oral jaw shape of loricariid catfishes. The broken stick method showed one axis was significant for the morphospace using traditional landmarks on the premaxilla, representing ~71% of the overall variation in shape. Species on the negative end of PC1 have a more elongate premaxilla, whereas species on the positive end had a squarer premaxilla (Fig. S1A). Traditional landmark methods did not effectively separate species from one another, with most species clustered on the positive end of PC1. The only exceptions were *Oxyropsis ephippia* and *Rhadinoloricaria macromystax* which fell on the negative end.

Three axes were significant for the morphospace using traditional landmarks on the maxilla. Principle component one accounted for ~31%, PC2 for ~27%, and PC3 for ~18% of shape variation (Fig S1C, S2). For all three axes the maxilla was less curved with smaller heads on the negative end and more curved with larger heads toward the positive end of the axes. There was less clustering of species within the morphospace; however, all subfamilies overlapped with one another across each axis.

The broken stick method showed three axes were significant for the morphospace using traditional landmarks on the lower jaw. PC1 accounted for ~48% of the variation, where the anguloarticular crests were taller with more elongated tooth cups on the negative end and shorter processes and tooth cups on positive end. PC2 accounted for ~14% of the variation in shape with more elongated tooth cups and shorter anguloarticulars on the negative end and shorter tooth cups and longer anguloarticulars on the positive end (Fig. S1E). The third PC accounted for ~12% of the shape variation where lower jaws with smaller processes fell to the negative end and jaws with taller processes were on the positive end (Fig. S3A). Within the morphospace there was a large cluster of species with three species falling to the negative end of PC1 (*Pareiorhaphis cameroni*, *Dentectus barbarmatus*, *Panaqolus* sp.) and two species on the positive end of PC1 (*Cordylancistrus torbesensis*, *Hisonotus maculipinnis*). All subfamilies overlapped with one another across each axis.

Automated landmarking was better able to separate species from one another. Although species were more separated from one another, there was no clear distinction between subfamilies in all automated landmarked morphospaces. For the premaxilla, the broken stick method found two significant axes, representing ~31% for PC1 and ~20% for PC2 (Fig. S1B). On the negative end of PC1, the premaxilla shape was more elongate and became squarer toward the positive end. The opposite was seen on PC2, where premaxilla shape was squarer toward the negative end and became more elongated toward the positive end.

For the maxilla, two significant axes were found. On PC1, ~36% of the overall shape variation was shown, where a more curved, club-like maxilla fell toward the negative end and a less curved, more stick-like maxilla fell on the positive end (Fig. S1D). PC2 accounted for ~12.6% of the shape variation where more curved bodies were placed on the negative end and becoming less curved toward the positive end.

The lower jaw had three significant axes representing about 75% of the total variation; PC1 at ~43%, PC2 at ~20%, and PC3 at ~12% (Figs. S1F, S3B). Across PC1, tooth cups became elongated with a more robust anguloarticular toward the positive end of the axis. On PC2, there were changes in the anguloarticular crest shape, with crests becoming taller toward the positive end. The PC3 axis primarily represented changes in the anguloarticular length, with shorter anguloarticulars toward the negative end and longer anguloarticulars toward the positive end. Although the lower jaw morphospace using automated landmarks was useful in separating species from one another, there were some misplacement of shape. Because the lower jaw could not be separated into the dentary and anguloarticular due to poor CT scanning quality and anatomical complexity, some species were misplaced in the morphospace. For example, *Cordylancistrus torbesensis*, *Pareiorhaphis cameroni*, and *Chaetostoma milesi* have long tooth cups and small anguloarticulars; however, these species are placed near species in the morphospace with short tooth cups and long anguloarticulars (*Loricariichthys maculatus* and *Dentectus barbarmatus*).

Phylomorphospaces. When the morphospace was trimmed to fit the phylogeny [34], the broken stick method found fewer significant axes for the traditional landmarked dataset of the lower jaw (2 significant axes), and the automated landmarked dataset for the lower jaw (2 significant axes).

### (B) Automated landmarking preforms better than traditional landmarking methods.

To compare how different landmarking schemes preformed to one another, we calculated the PLS between traditional and automated methods (Fig. S15). An r-PLS score of 1 suggests the two datasets are the same, whereas a score of 0 would suggest the datasets are completely different from one another. The traditional and automated landmarks for combined shape showed that traditional and automated landmarking preformed similarly with an r-PLS = 0.942 (*p* = 0.001; Fig. S15A). However, when traditional and automated landmarking of induvial bones were compared to one another, there was more variation between datasets, with the premaxilla scoring a r-PLS of 0.844 (*p* = 0.005), the maxilla a r-PLS of 0.75 (*p* = 0.032), and the lower jaw a r-PLS of 0.923 (*p* = 0.001; Fig. S15B-D). In addition to differences between the datasets, the warped meshes for traditional landmarking failed to capture realistic shapes, whereas the automated landmarks produced warped meshes that looked representative of loricariid jaws (Fig. S5-7, S11-13).
