# Supplementary figures and images for: Chew on this: Oral jaw shape is not correlated with diet type in loricariid catfishes
Source: PLoS One. 2022 Nov 2;17(11):e0277102. doi: 10.1371/journal.pone.0277102 (PMC9629652; doi:10.1371/journal.pone.0277102)

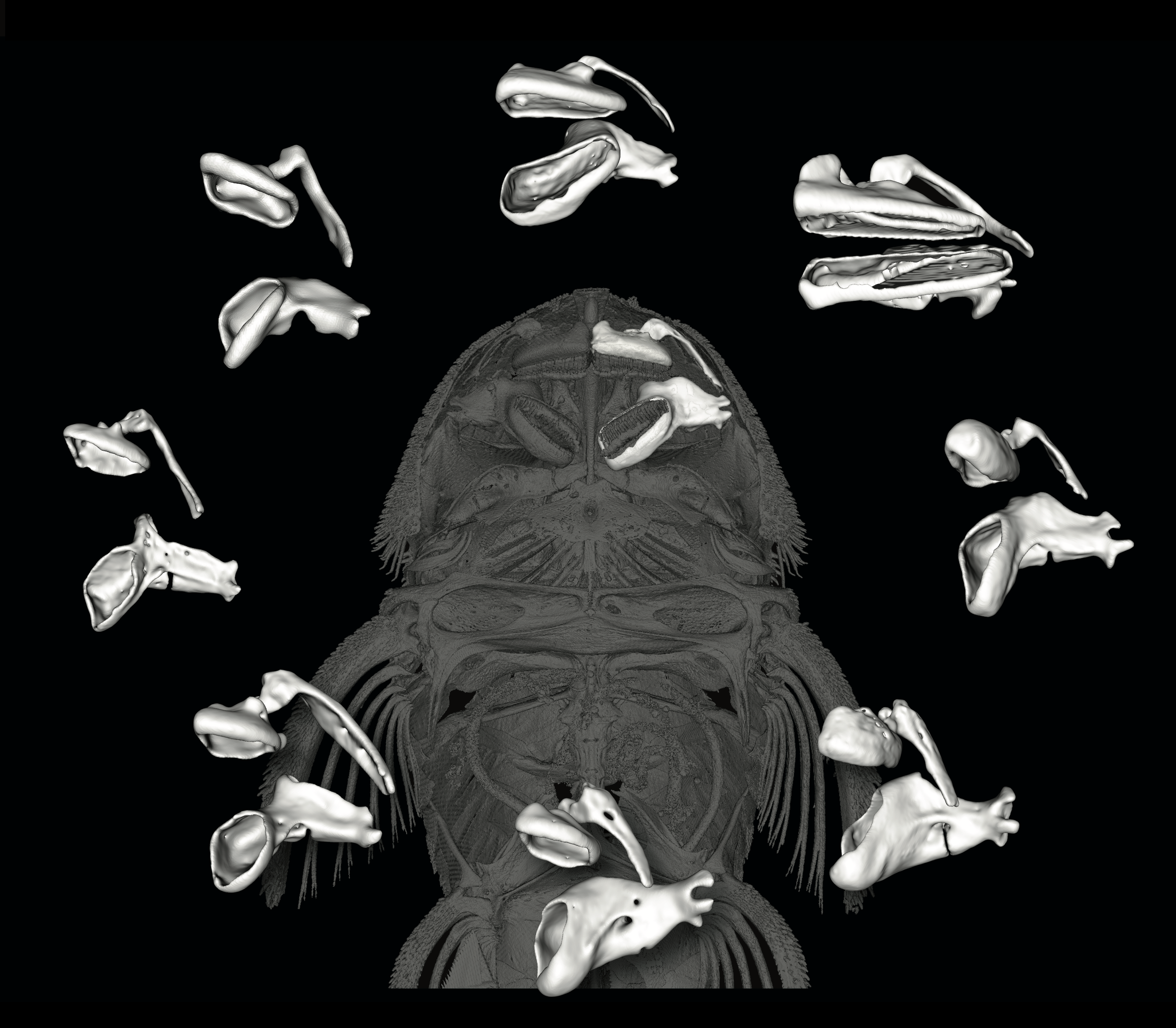

Supplement: S1 File — (TIF) [file pone.0277102.s002.tif]

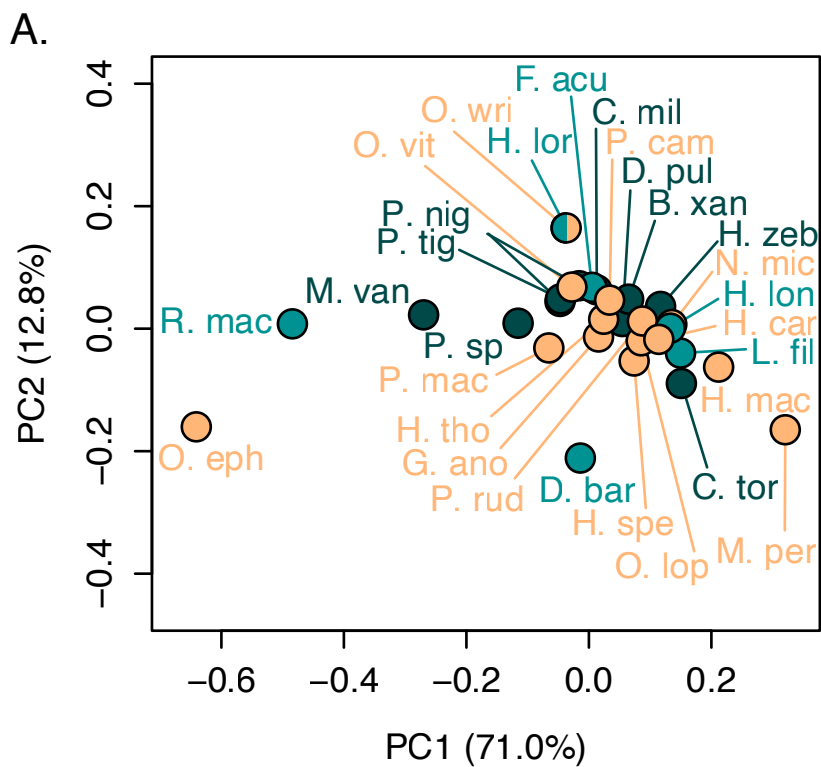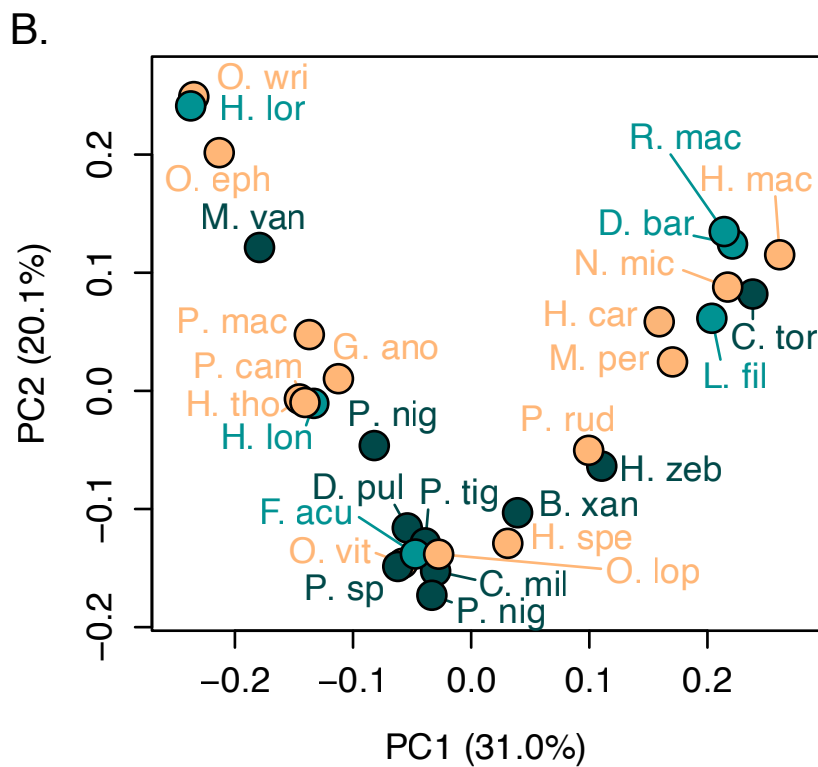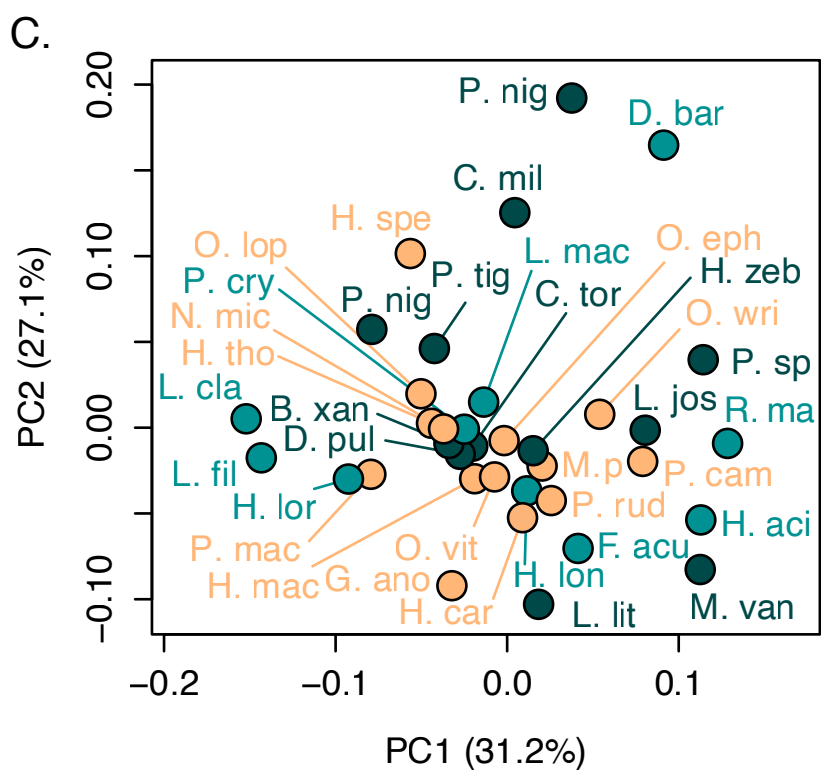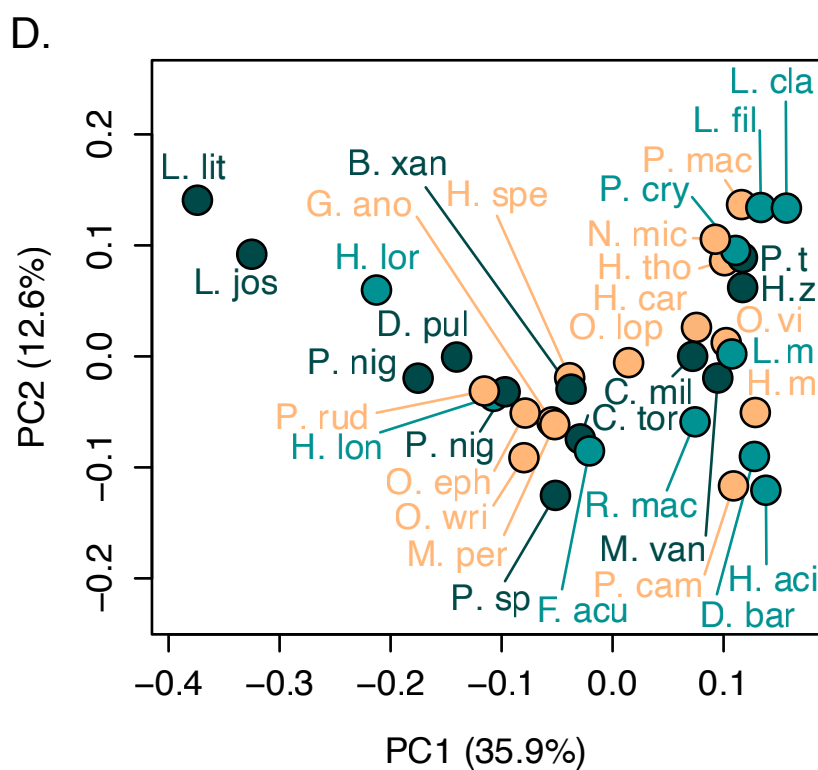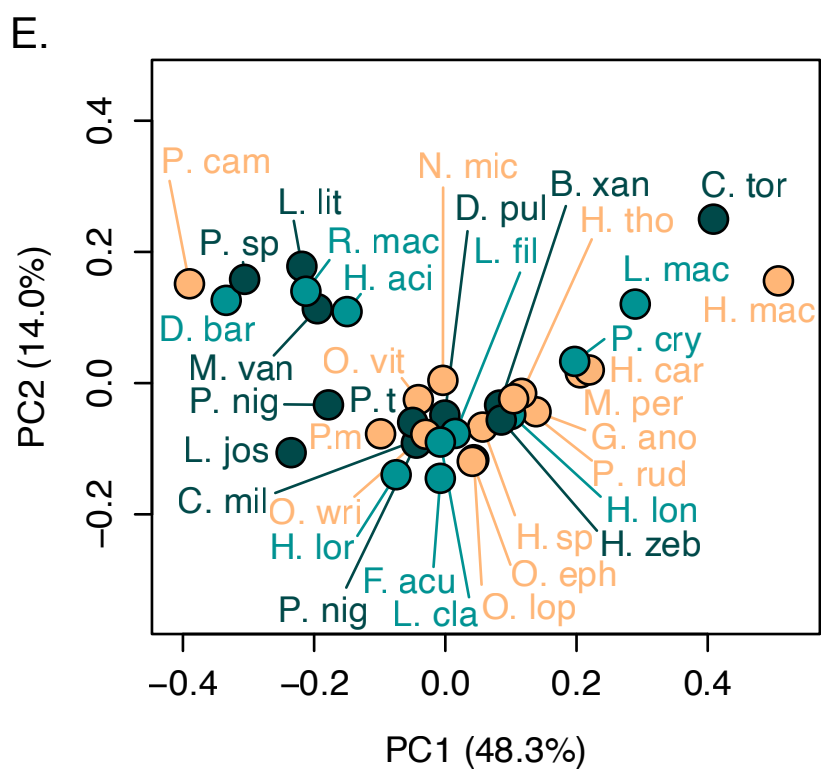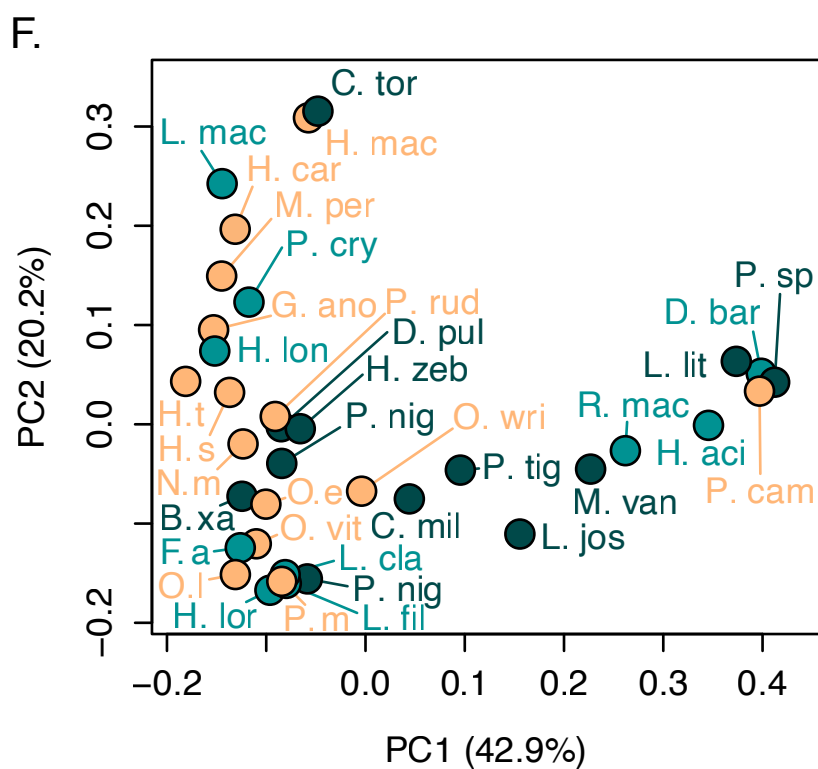

Supplement: S1 Fig — Morphospaces for individual bones using (A) traditional and (B) automated landmarks on the premaxilla, (C) traditional and (D) automated landmarks on the maxilla, and (E) traditional and (F) automated landmarks on the lower jaw. Subfamilies denoted by colors; Hypoptopomatinae in orange, Hypostominae in dark green, Loricariinae in light green, and Neoplecostominae in pink. (PDF) [file pone.0277102.s006.pdf]

PC 3: 17.66%

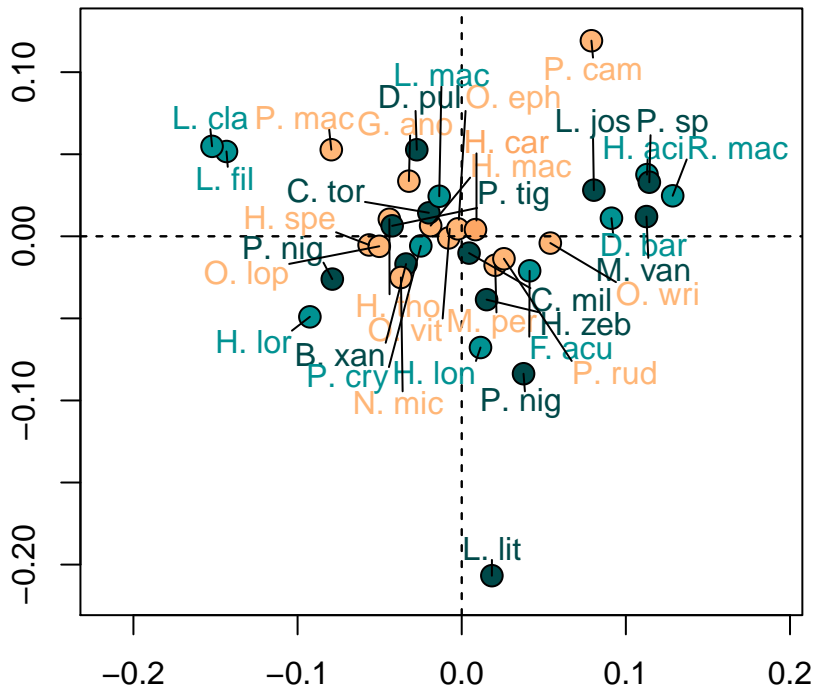

PC 1: 31.17%

Supplement: S2 Fig — Subfamilies denoted by colors; Hypoptopomatinae in orange, Hypostominae in dark green, Loricariinae in light green, and Neoplecostominae in pink. (PDF) [file pone.0277102.s007.pdf]

A.

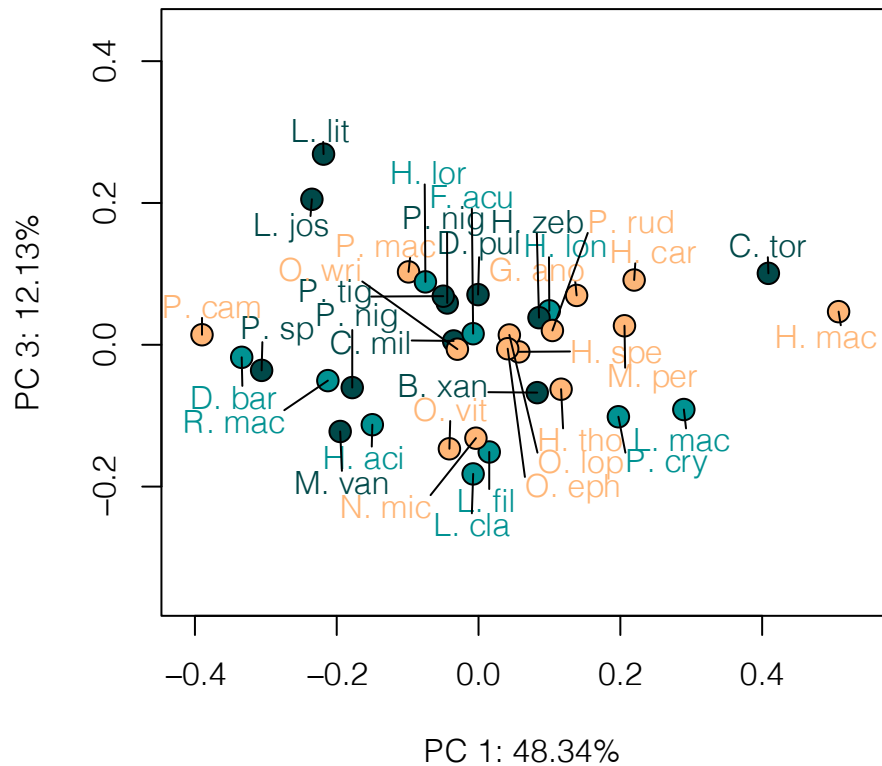

B.

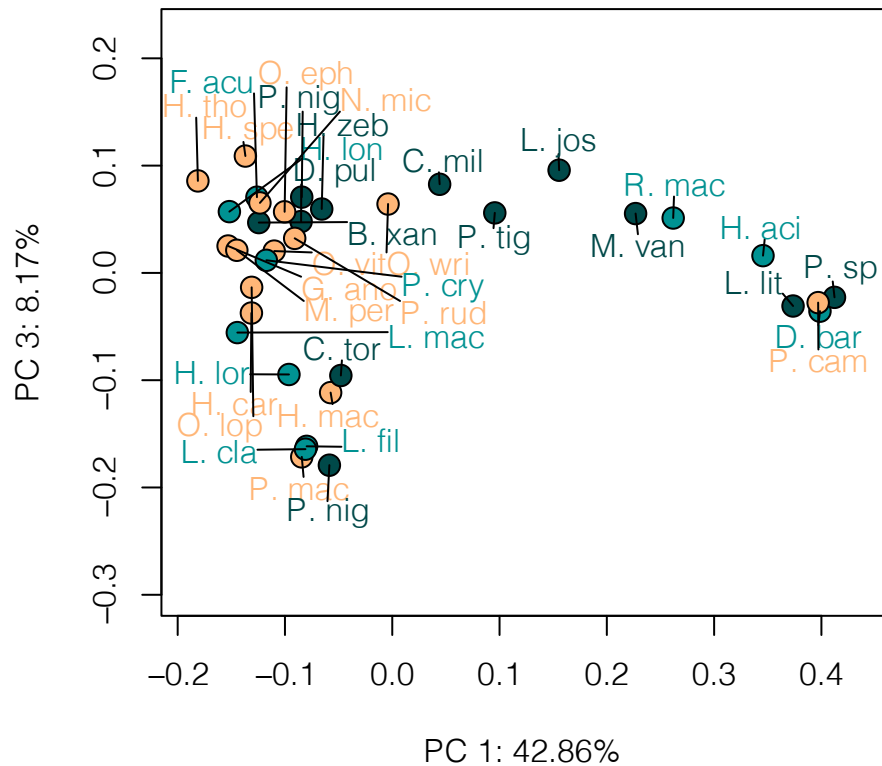

Supplement: S3 Fig — Morphospace for the lower jaw using (A) traditional landmarks and (B) automated landmarking methods for PC1 and PC3. Subfamilies denoted by colors; Hypoptopomatinae in orange, Hypostominae in dark green, Loricariinae in light green, and Neoplecostominae in pink. (PDF) [file pone.0277102.s008.pdf]

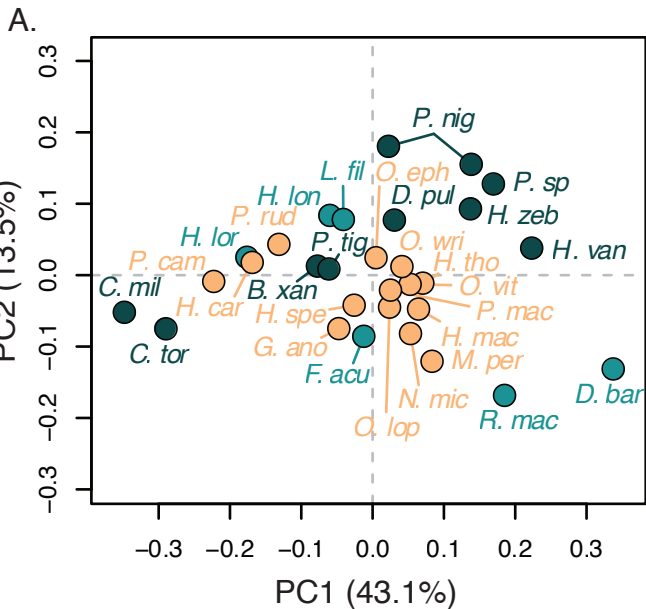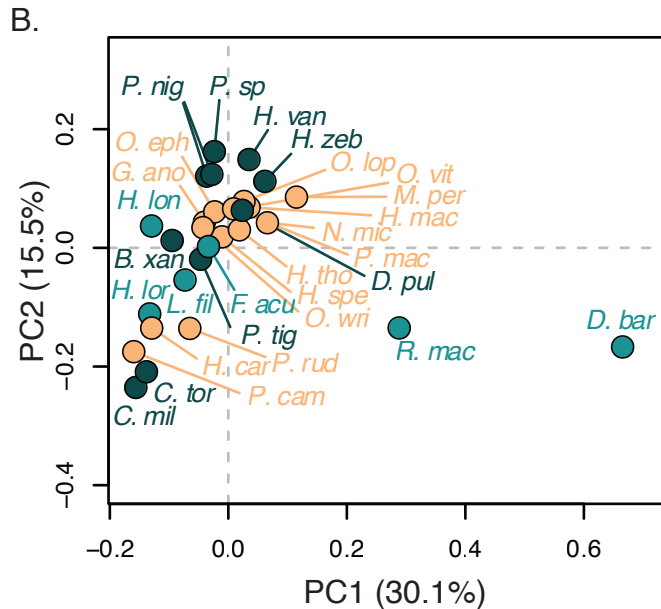

Supplement: S4 Fig — Morphospaces for the combine shape (premaxilla, maxilla, and lower jaw) for loricariid species using (A) traditional and (B) automated landmarking. Subfamilies denoted by colors; Hypoptopomatinae in orange, Hypostominae in dark green, Loricariinae in light green, and Neoplecostominae in pink. (PDF) [file pone.0277102.s009.pdf]

# Traditional Landmarks

# Automated Landmarks

(-)

(+)

(-)

(+)

PC1

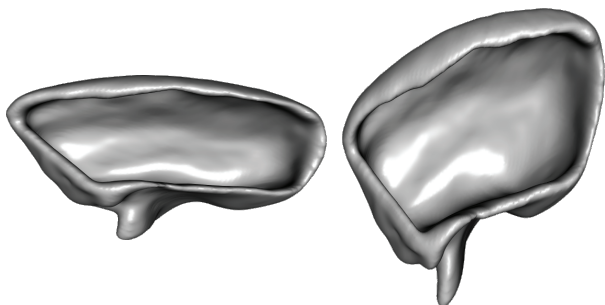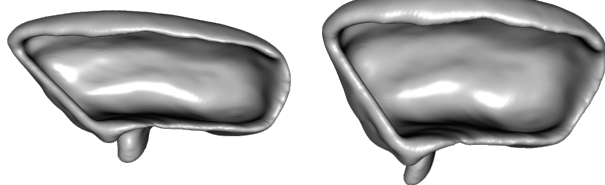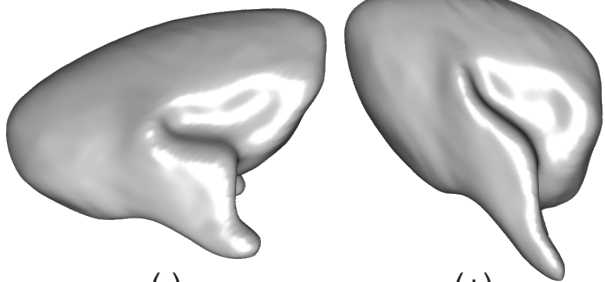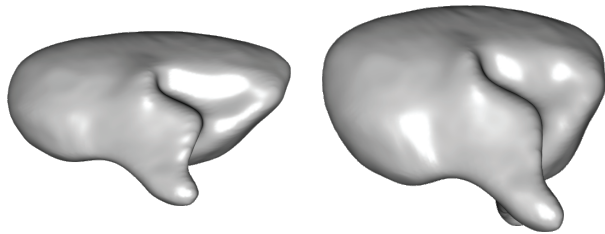

(-)

(+)

(-)

(+)

PC2

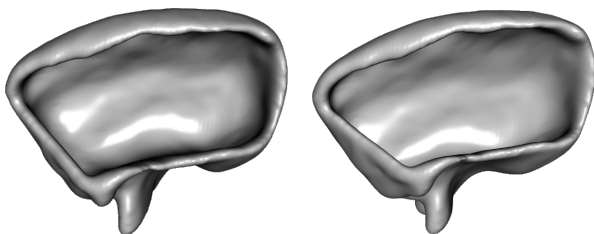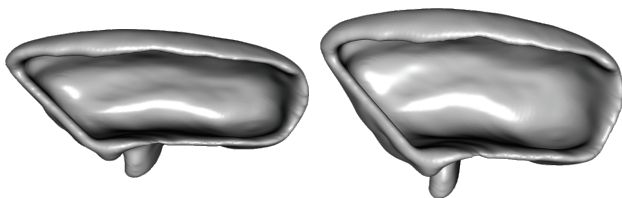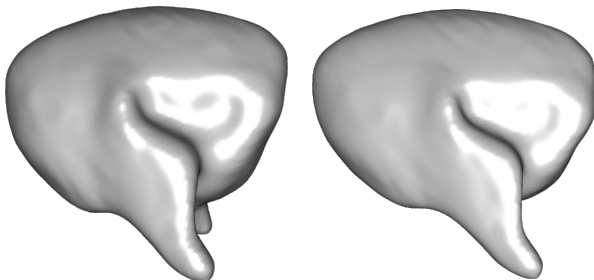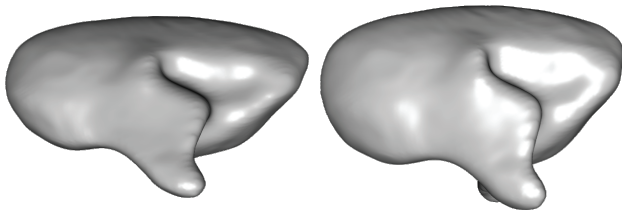

(-)

(+)

PC3

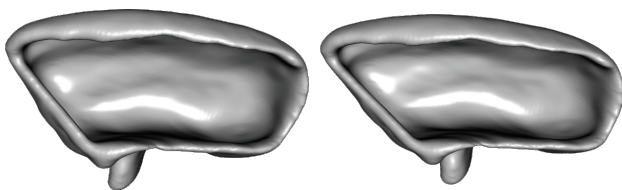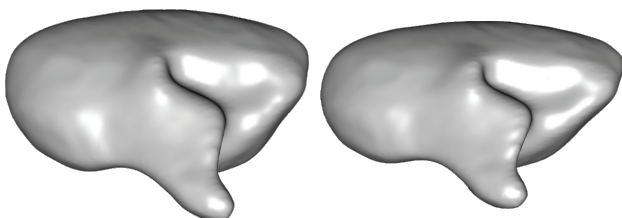

Supplement: S5 Fig — (PDF) [file pone.0277102.s010.pdf]

## Traditional Landmarks

## Automated Landmarks

(-)

(+)

(-)

(+)

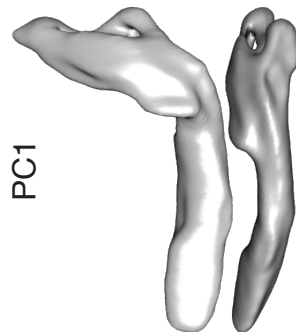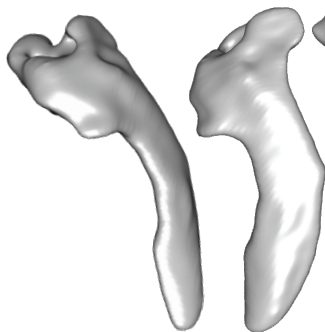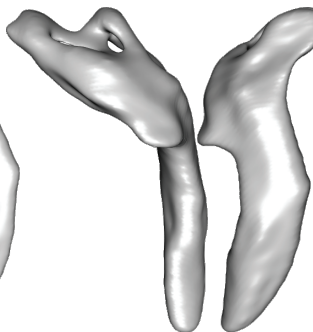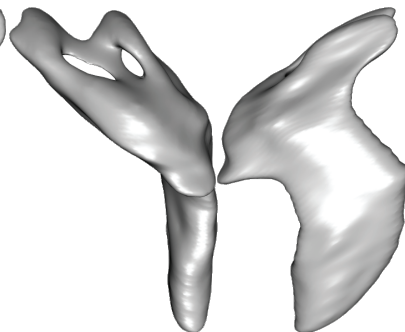

PC1

(-)

(+)

(-)

(+)

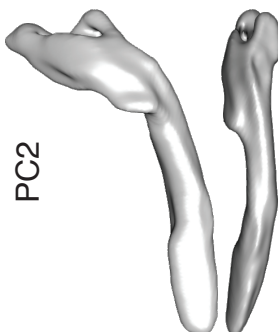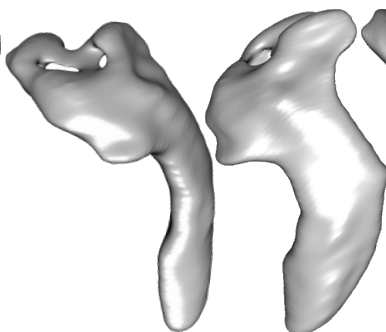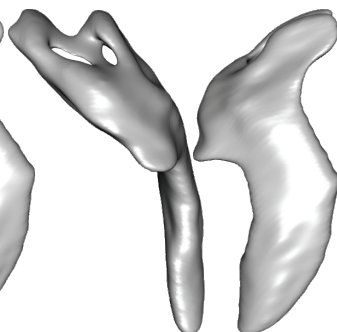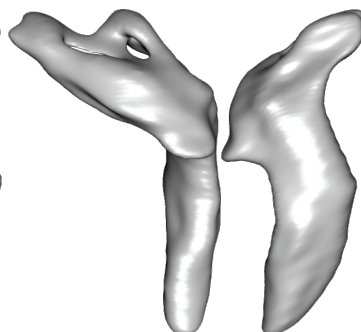

PC2

(-)

(+)

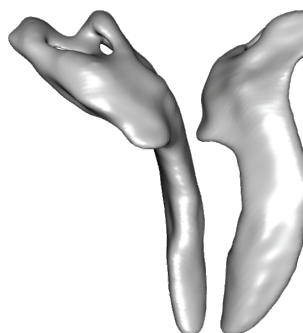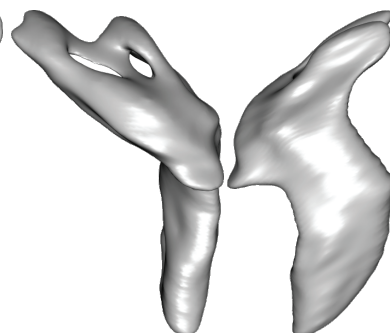

PC3

Supplement: S6 Fig — (PDF) [file pone.0277102.s011.pdf]

## Traditional Landmarks

## Automated Landmarks

(-)

(+)

(-)

(+)

PC1

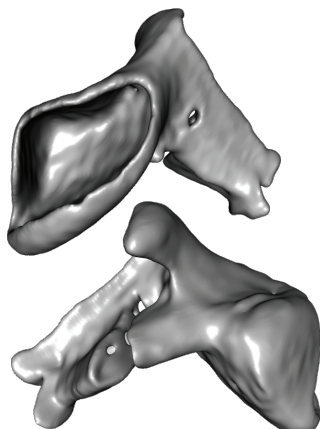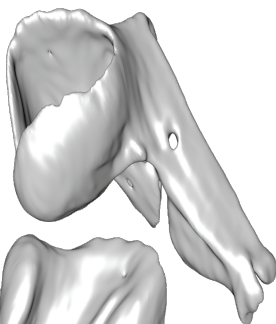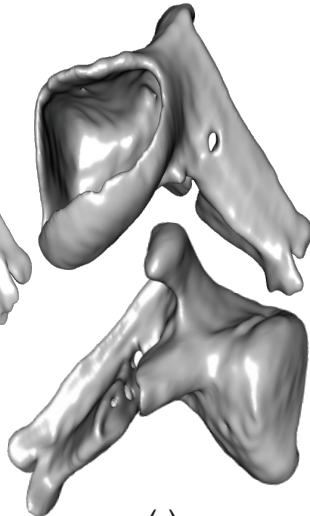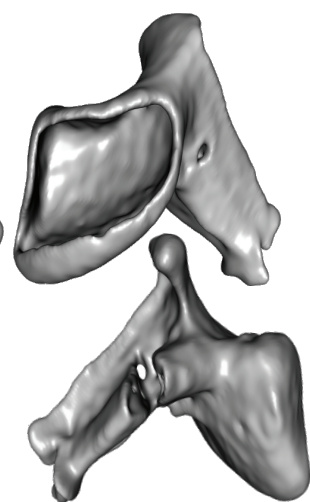

(-)

(+)

(-)

(+)

PC2

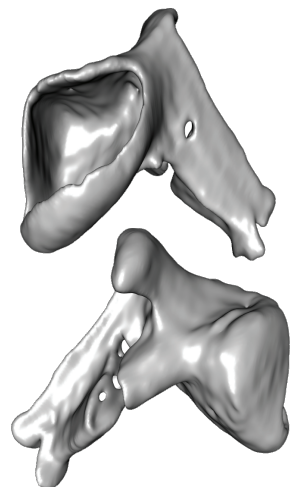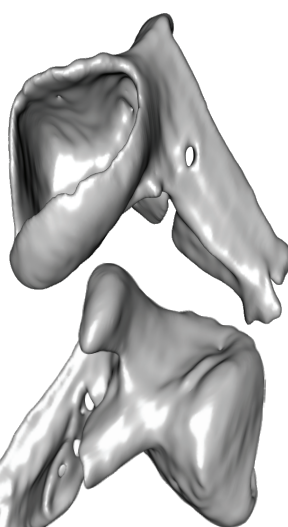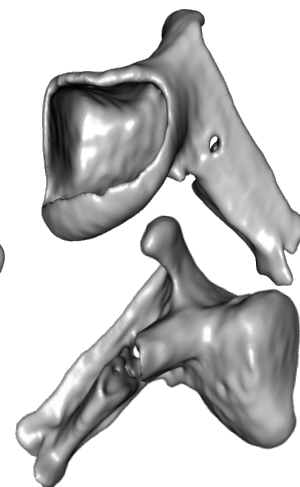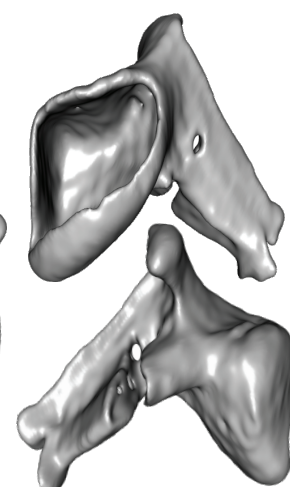

(-)

(+)

PC3

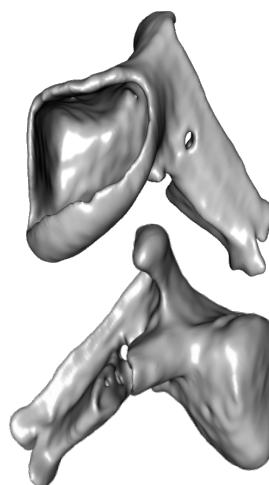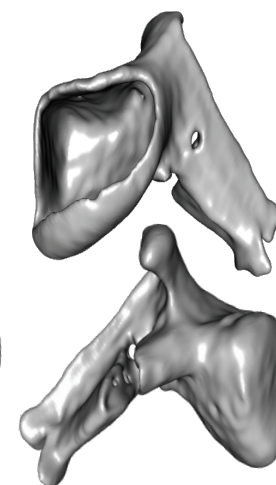

Supplement: S7 Fig — (PDF) [file pone.0277102.s012.pdf]

PC 3: 11.49%

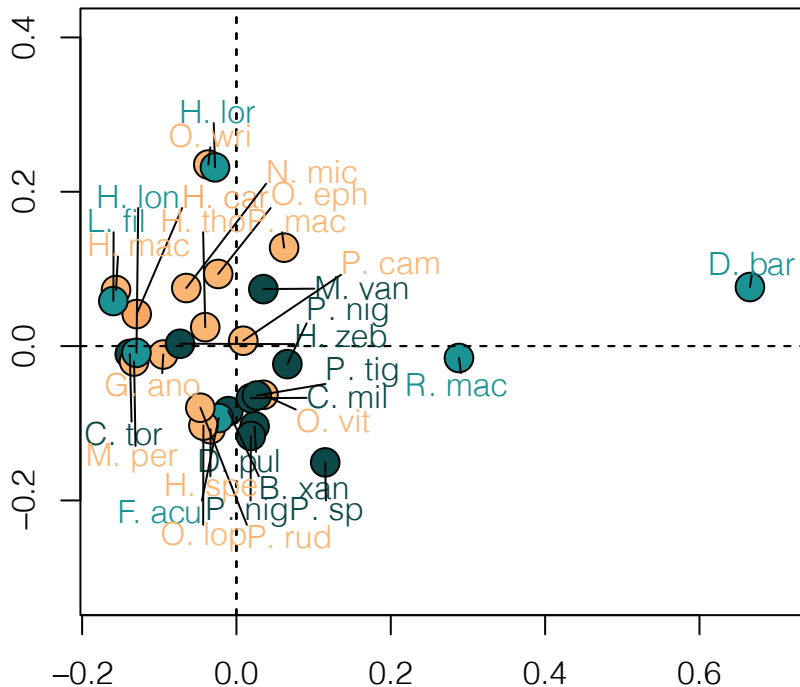

PC 1: 30.06%

Supplement: S8 Fig — Subfamilies denoted by colors; Hypoptopomatinae in orange, Hypostominae in dark green, Loricariinae in light green, and Neoplecostominae in pink. (PDF) [file pone.0277102.s013.pdf]

A.

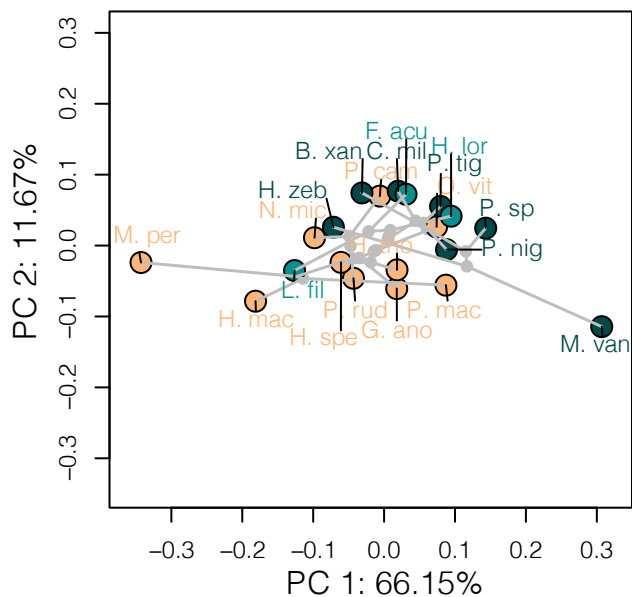

B.

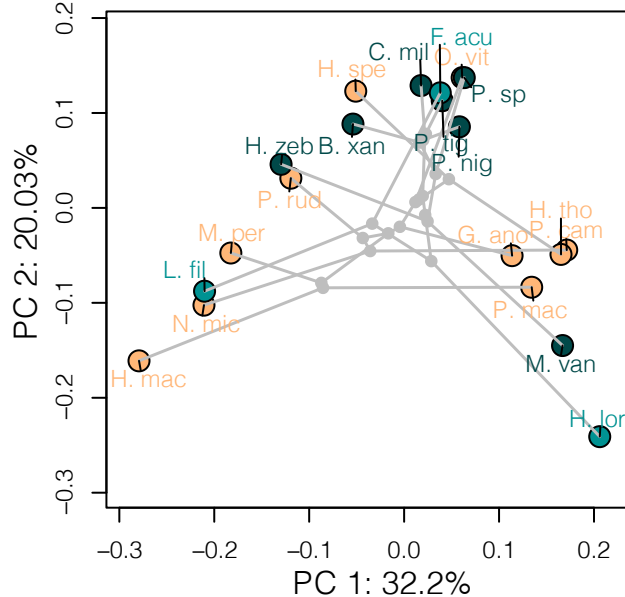

C.

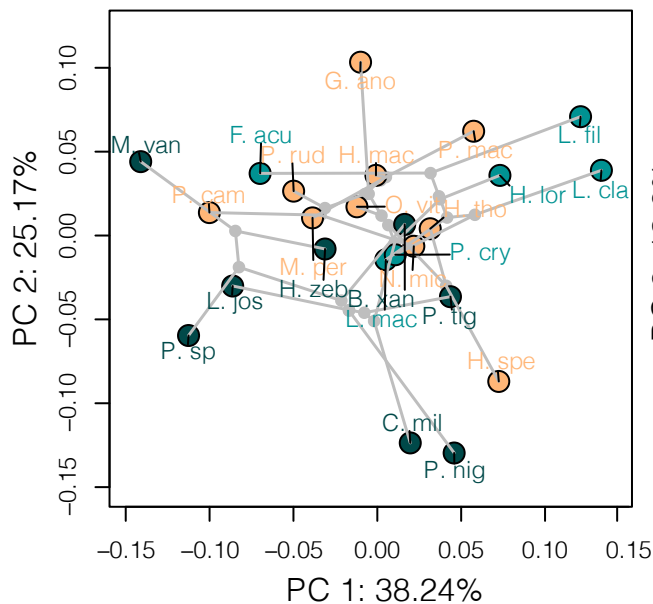

D.

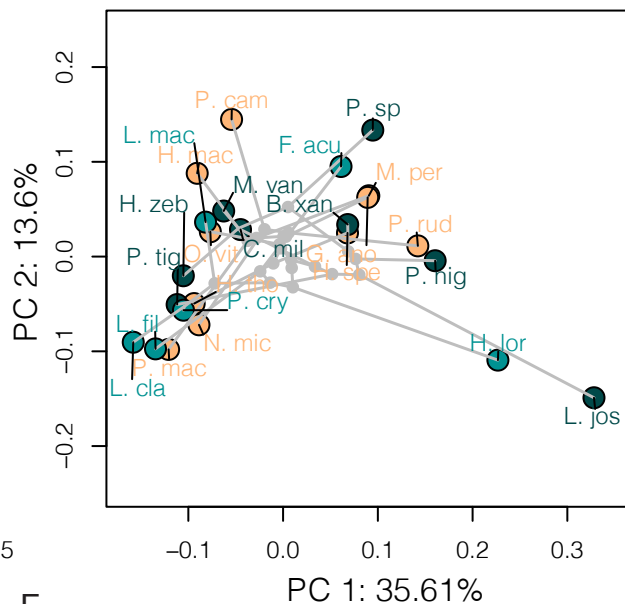

E.

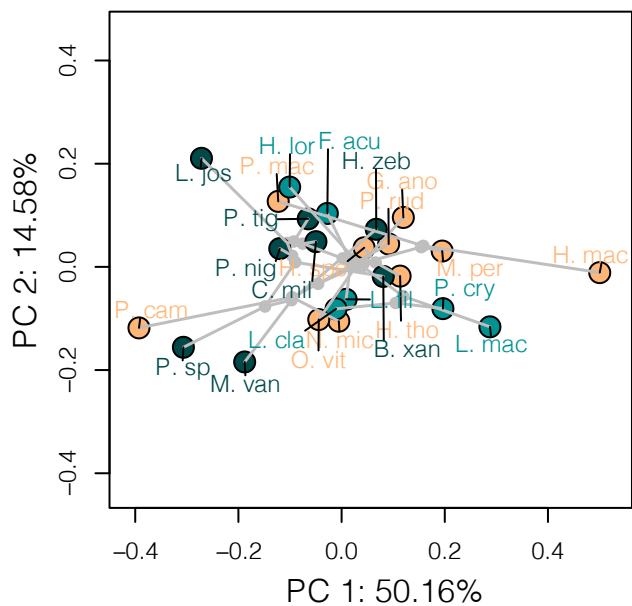

F.

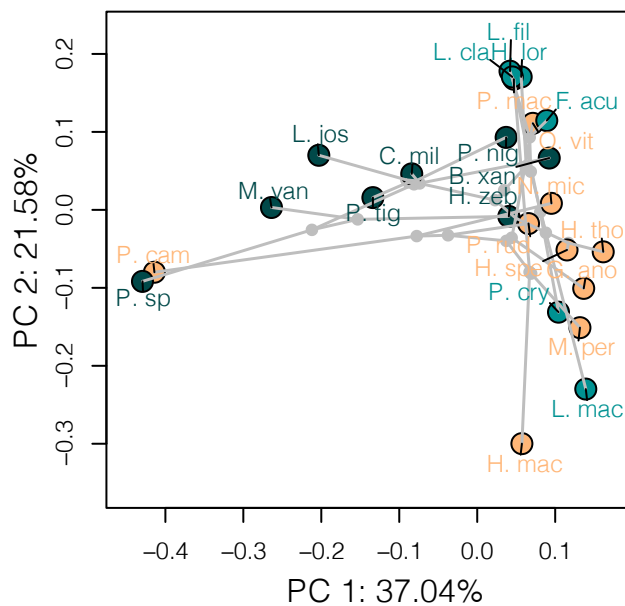

Supplement: S9 Fig — Phylomorphospaces for individual bones using (A) traditional and (B) automated landmarks on the premaxilla, (C) traditional and (D) automated landmarks on the maxilla, and (E) traditional and (F) automated landmarks on the lower jaw. Subfamilies denoted by colors; Hypoptopomatinae in orange, Hypostominae in dark green, and Loricariinae in light green. (PDF) [file pone.0277102.s014.pdf]

PC 3: 12.07%

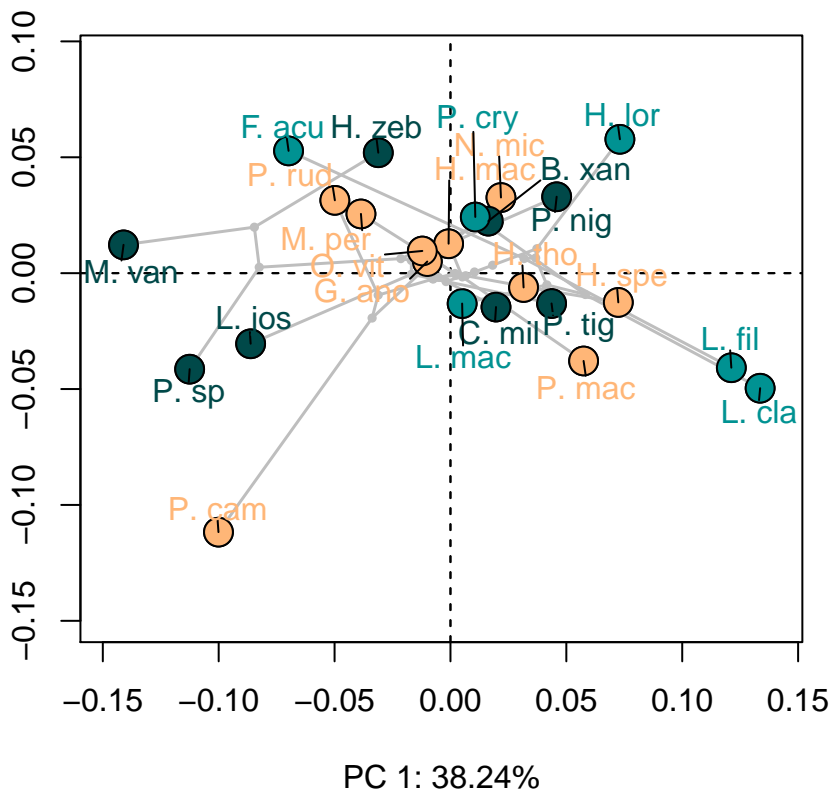

Supplement: S10 Fig — Subfamilies denoted by colors; Hypoptopomatinae in orange, Hypostominae in dark green, and Loricariinae in light green. (PDF) [file pone.0277102.s015.pdf]

# Traditional Landmarks

# Automated Landmarks

(-)

(+)

(-)

(+)

PC1

PC2

PC3

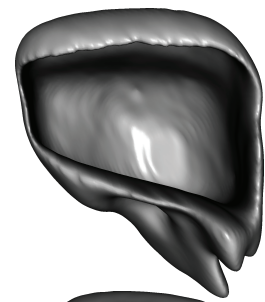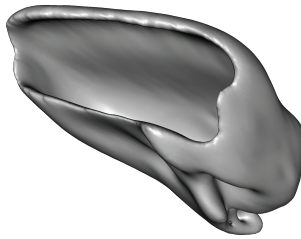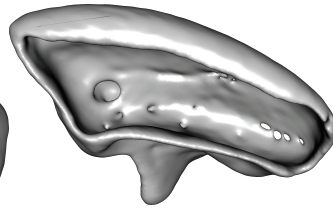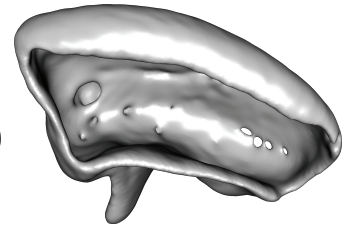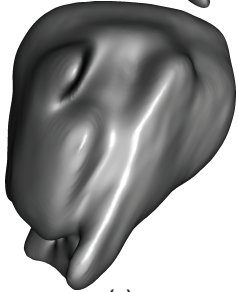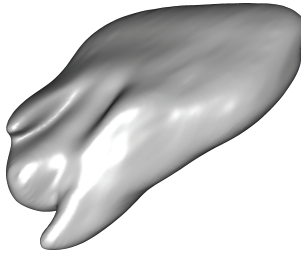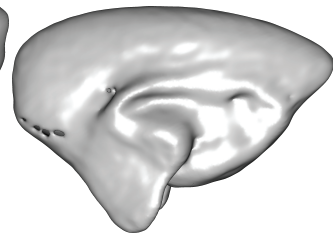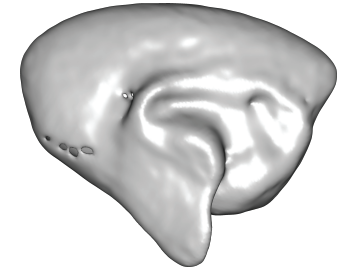

(-)

(+)

(-)

(+)

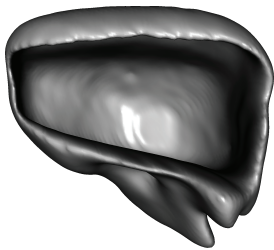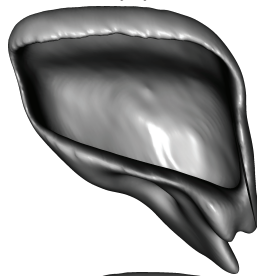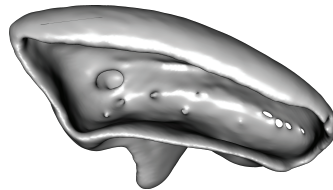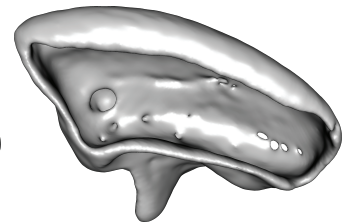

(-)

(+)

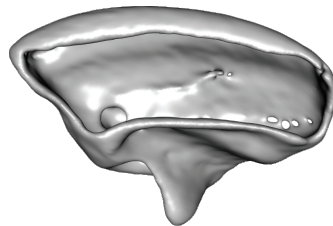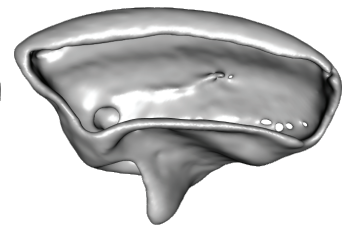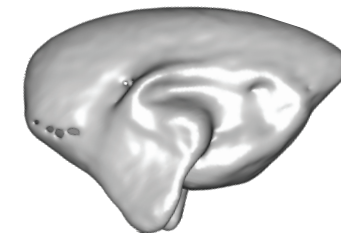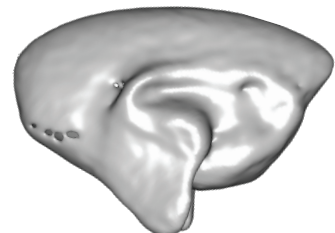

Supplement: S11 Fig — Phylomorphospace for the combine shape (premaxilla, maxilla, and lower jaw) of loricariid species using automated landmarking. Subfamilies denoted by colors; Hypoptopomatinae in orange, Hypostominae in dark green, and Loricariinae in light green. (PDF) [file pone.0277102.s016.pdf]

## Traditional Landmarks

(-)

(+)

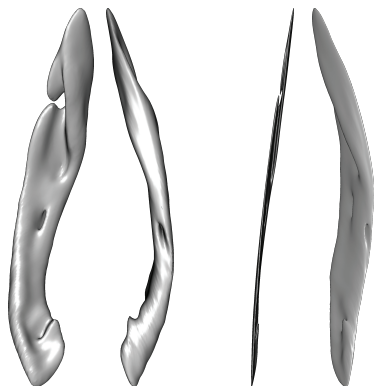

(-)

(+)

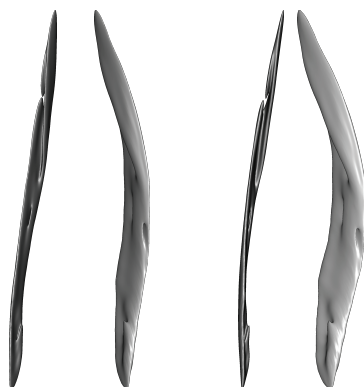

## Automated Landmarks

(-)

(+)

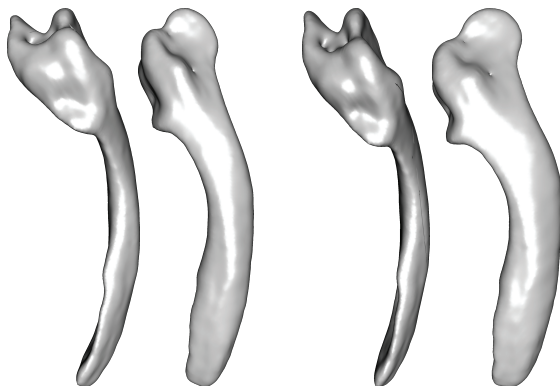

(-)

(+)

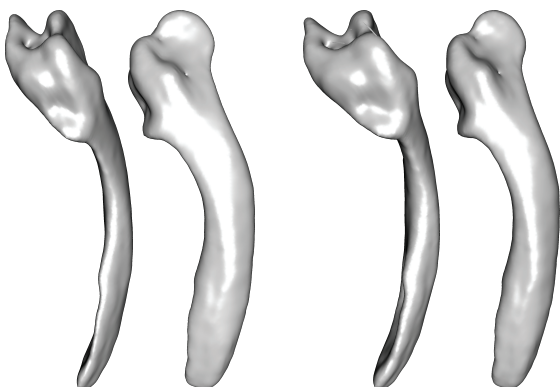

(-)

(+)

PC3

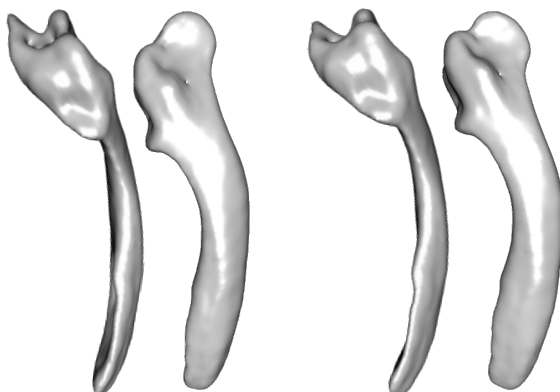

PC1

PC2

Supplement: S12 Fig — (PDF) [file pone.0277102.s017.pdf]

## Traditional Landmarks

(-)

(+)

## Automated Landmarks

(-)

(+)

PC1

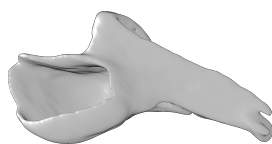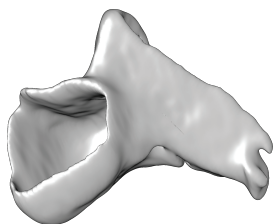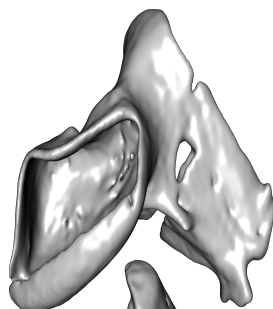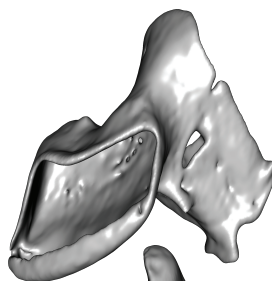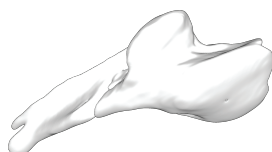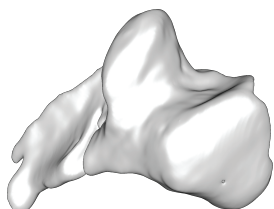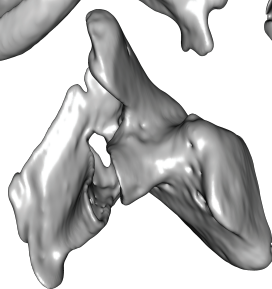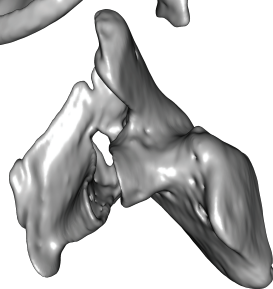

(-)

(+)

(-)

(+)

PC2

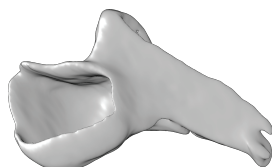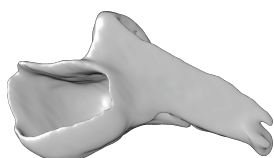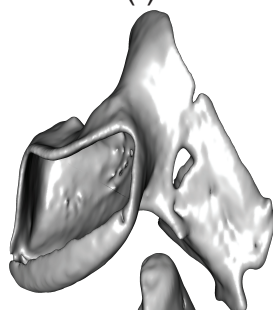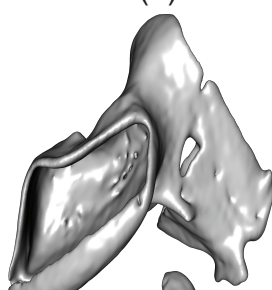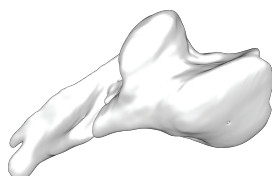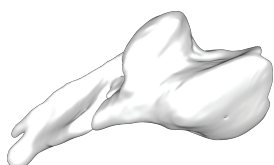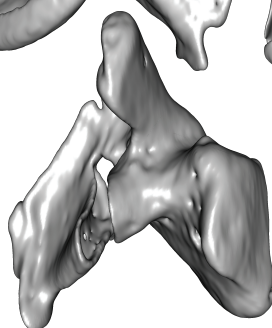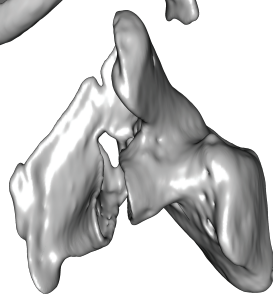

(-)

(+)

PC3

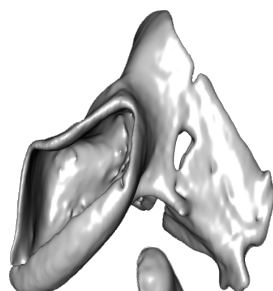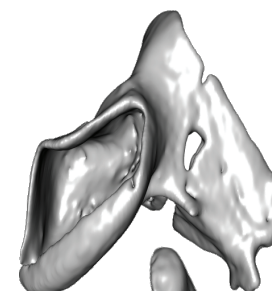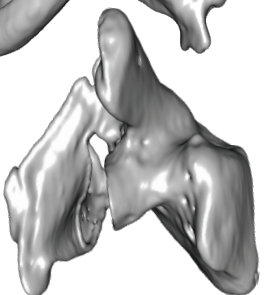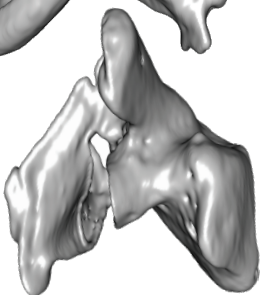

Supplement: S13 Fig — (PDF) [file pone.0277102.s018.pdf]

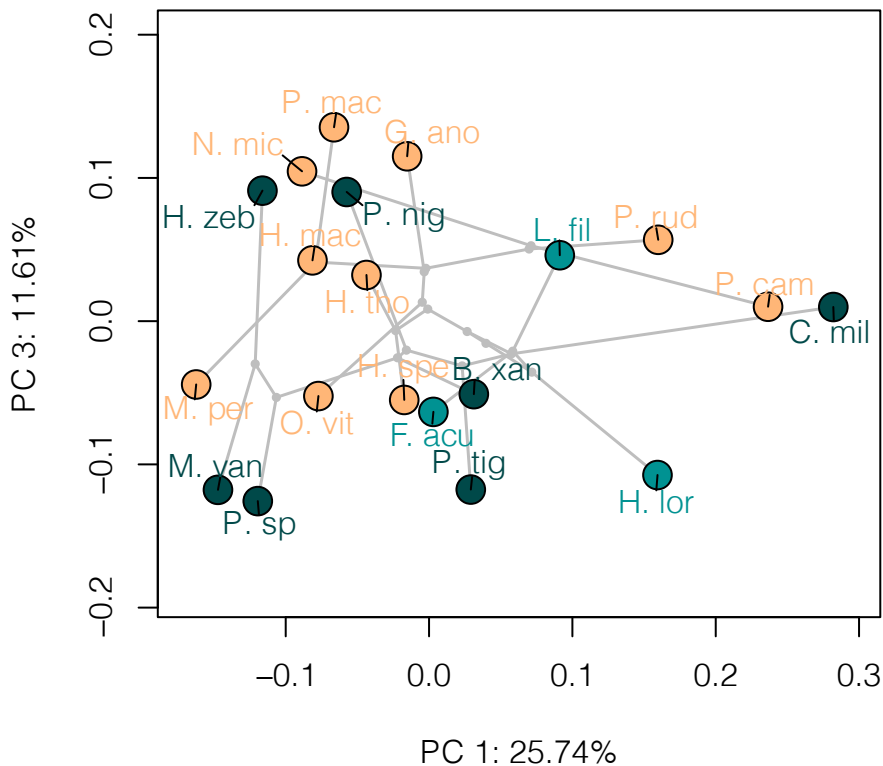

Supplement: S14 Fig — (PDF) [file pone.0277102.s019.pdf]

A.

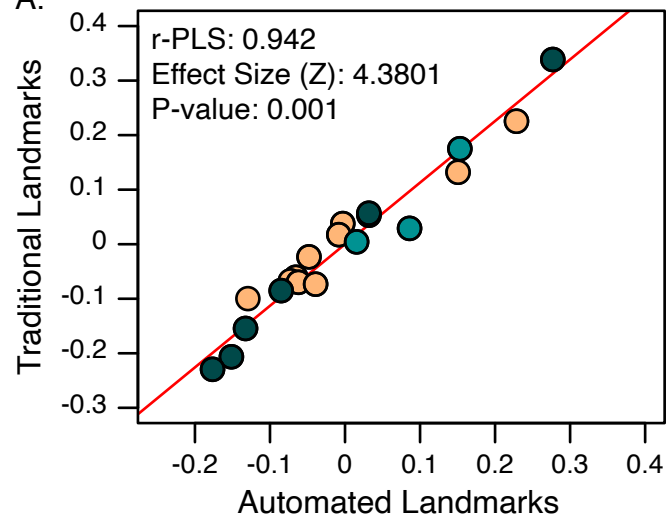

B.

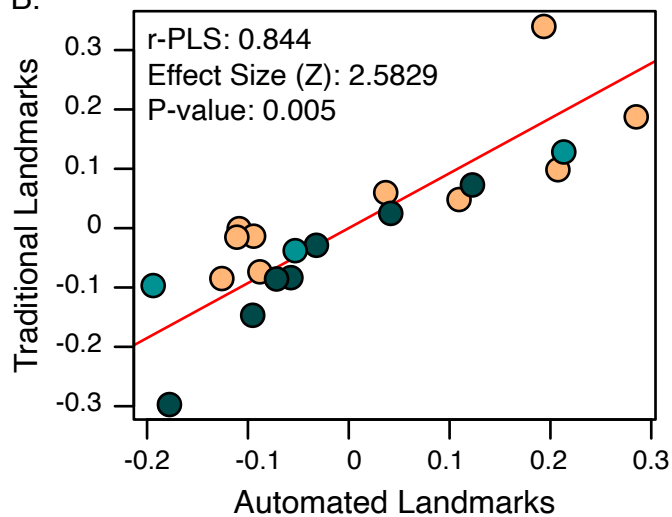

C.

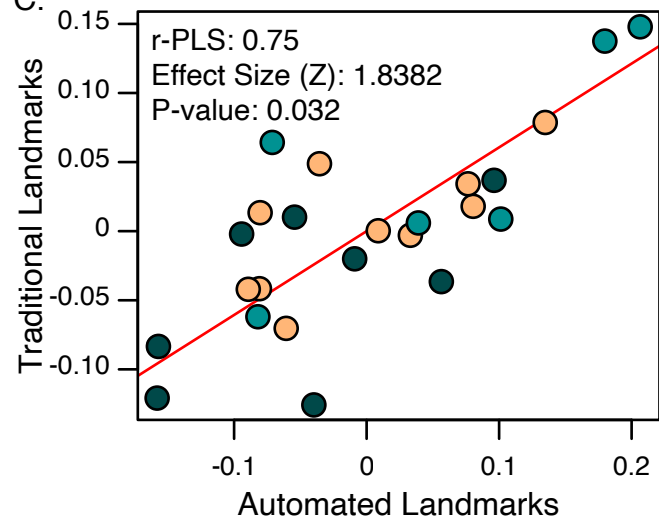

D.

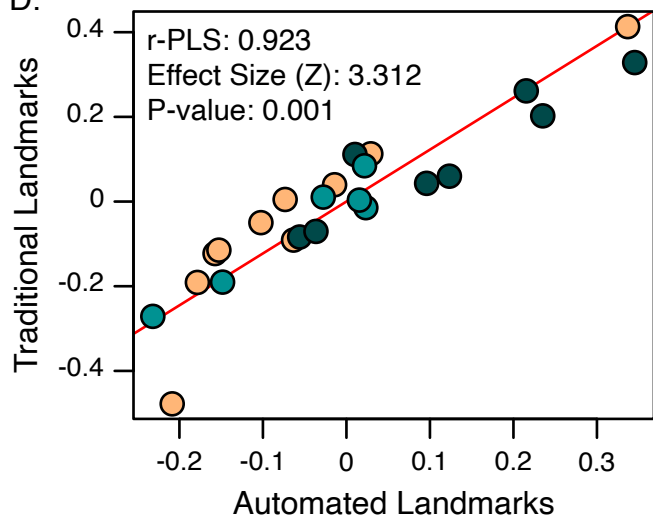

Supplement: S15 Fig — Partial least squares (PLS) comparing the traditional landmarking skeme to automated landmarking methods for (A) combine oral jaw shape, (B) the premaxilla, (C) the maxilla, and (D) the lower jaw for loricariid catfishes. Subfamilies denoted by colors; Hypoptopomatinae in orange, Hypostominae in dark green, and Loricariinae in light green. (PDF) [file pone.0277102.s020.pdf]

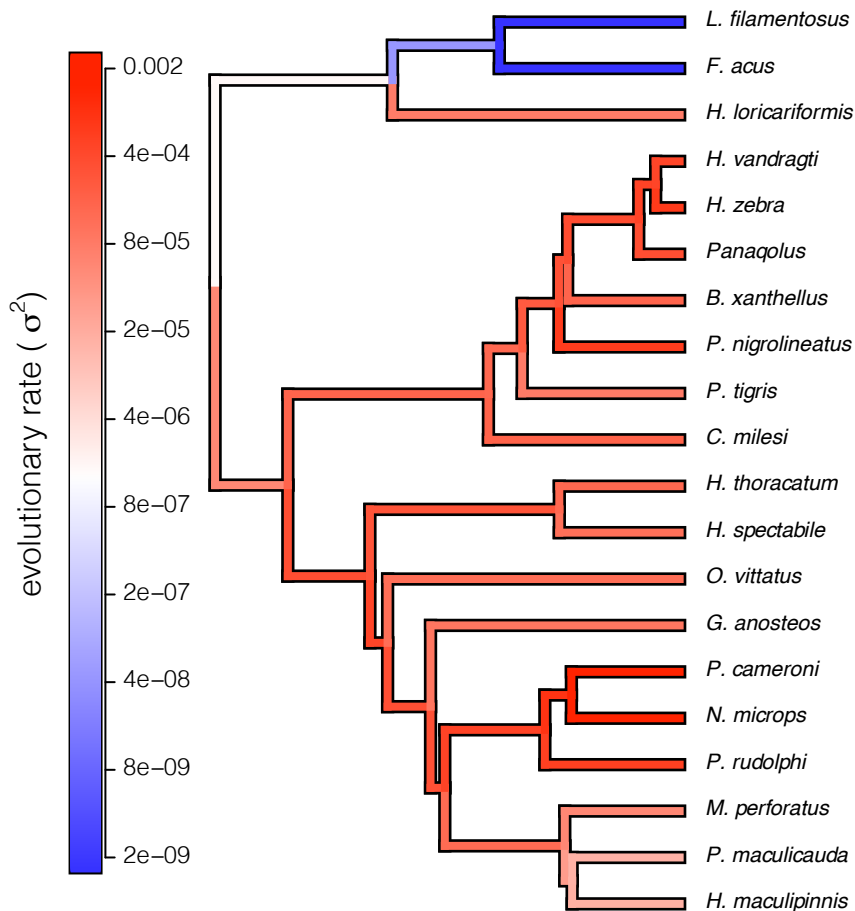

Supplement: S16 Fig — (PDF) [file pone.0277102.s021.pdf]
